# The Effectiveness of Cognitive Behavioural Treatment for Non-Specific Low Back Pain: A Systematic Review and Meta-Analysis

**DOI:** 10.1371/journal.pone.0134192

**Published:** 2015-08-05

**Authors:** Helen Richmond, Amanda M. Hall, Bethan Copsey, Zara Hansen, Esther Williamson, Nicolette Hoxey-Thomas, Zafra Cooper, Sarah E Lamb

**Affiliations:** 1 Centre for Rehabilitation Research, Nuffield Department of Orthopaedics Rheumatology and Musculoskeletal Sciences, University of Oxford, Oxford, England, United Kingdom; 2 The George Institute for Global Health, University of Oxford, Oxford, England, United Kingdom; 3 Department of Psychiatry, Medical Sciences Division, Warneford Hospital, Oxford, United Kingdom; University of North Carolina at Chapel Hill, UNITED STATES

## Abstract

**Objectives:**

To assess whether cognitive behavioural (CB) approaches improve disability, pain, quality of life and/or work disability for patients with low back pain (LBP) of any duration and of any age.

**Methods:**

Nine databases were searched for randomised controlled trials (RCTs) from inception to November 2014. Two independent reviewers rated trial quality and extracted trial data. Standardised mean differences (SMD) and 95% confidence intervals were calculated for individual trials. Pooled effect sizes were calculated using a random-effects model for two contrasts: CB versus no treatment (including wait-list and usual care (WL/UC)), and CB versus other guideline-based active treatment (GAT).

**Results:**

The review included 23 studies with a total of 3359 participants. Of these, the majority studied patients with persistent LBP (>6 weeks; n=20). At long term follow-up, the pooled SMD for the WL/UC comparison was -0.19 (-0.38, 0.01) for disability, and -0.23 (-0.43, -0.04) for pain, in favour of CB. For the GAT comparison, at long term the pooled SMD was -0.83 (-1.46, -0.19) for disability and -0.48 (-0.93, -0.04) for pain, in favour of CB. While trials varied considerably in methodological quality, and in intervention factors such as provider, mode of delivery, dose, duration, and pragmatism, there were several examples of lower intensity, low cost interventions that were effective.

**Conclusion:**

CB interventions yield long-term improvements in pain, disability and quality of life in comparison to no treatment and other guideline-based active treatments for patients with LBP of any duration and of any age.

**Systematic Review Registration:**

PROSPERO protocol registration number: CRD42014010536.

## Introduction

Non-specific Low Back Pain (LBP) causes more disability globally than any other condition [1]. Recent estimates suggest that 20–56% of adults will experience LBP within a single year and that most people will experience LBP at some point during their lives [1]. In the UK, the financial burden of LBP is estimated to be £2.8 billion per annum in direct costs alone [2]. Therefore, the effective management of LBP is a major concern for the individual, the economy and society as a whole [3,4]. Recommended treatments include education, exercise, manual therapy and acupuncture [3]. However, there is insufficient evidence that these treatments provide long term functional improvements and evidence suggests that one is not superior to another [5–8]. More recently, Cognitive Behavioural (CB) interventions for LBP have been growing in popularity [9–11] and are one of the most cost-effective treatments available for LBP to date [12].

A CB intervention refers to a form of psychological treatment that uses cognitive and behavioural techniques drawn from evidence-based models [13]. Sometimes referred to as a ‘family of treatments’ as there are specific forms of CB interventions for different health problems, they share basic common elements. Cognitive and behavioural techniques target features that are thought to be maintaining an illness/disability, namely distorted cognitions and maladaptive behaviours [13]. While the actual mechanisms underlying the effectiveness of CB techniques are not well understood [13,14], theoretical models suggest that symptoms can be improved through the modification of these cognitions and behaviours [13].

The National Institute for Health and Care Excellence (NICE) guideline for the management of persistent non-specific LBP stipulates that there is inconclusive evidence regarding the effectiveness of CB interventions for persistent non-specific LBP [3]. Since the publication of these guidelines, there has been increasing empirical evidence supporting the use of CB treatment strategies for the management of persistent LBP [6,9–11]. It is therefore timely to review the evidence on the use of CB interventions for the management of LBP.

Previous systematic reviews of CB interventions for LBP have excluded studies with older adults (over 65 years of age) [15–18] and patients with pain less than 12 weeks in duration. This limits the generalisability of the findings to broader populations. For example, recent research suggests that LBP is most prevalent in later life [19] and that there is an increased risk of chronicity if symptoms persist after 4–6 weeks [20–22]. Therefore, the aim of this review was to provide an up-to-date synthesis of the evidence regarding the effectiveness of CB interventions for the management of non-specific LBP, and to ensure eligibility criteria that would allow inclusion of trials of older people and LBP of any duration.

## Materials and Methods

The primary objective was to assess the effectiveness of CB interventions in comparison to no treatment and other conservative guideline active treatments, on pain, disability and quality of life in adults with non-specific LBP. While we assessed short-term (ST) (as close to 6 weeks and not exceeding 12 weeks) effects, our primary end point of interest was long-term (LT) (closest to 52 weeks and >26weeks). This review followed a protocol registered on PROSPERO (reference: CRD42014010536).

### Data sources and searches

Using search terms from the Cochrane Back Review Group (CBRG, 2013b) (S1 Fig Search strategy), a sensitive search of 9 electronic databases (Cochrane Central Register of Controlled Trials (CENTRAL), MEDLINE (1966 to date), EMBASE (1988 to date), CINAHL (1982 to date), AMED (1985 to date), Physiotherapy Evidence Database (PEDro), the Cochrane Back Review Group (CBRG) Trials Register, PsycINFO and OpenGrey (www.opengrey.eu) was performed from inception to November 2014. In addition, searches of reference lists of all included studies and relevant systematic reviews as well as personal communication was undertaken to identify potentially eligible studies.

### Selections of studies and data extraction

#### Inclusion criteria

**Fig 1:**
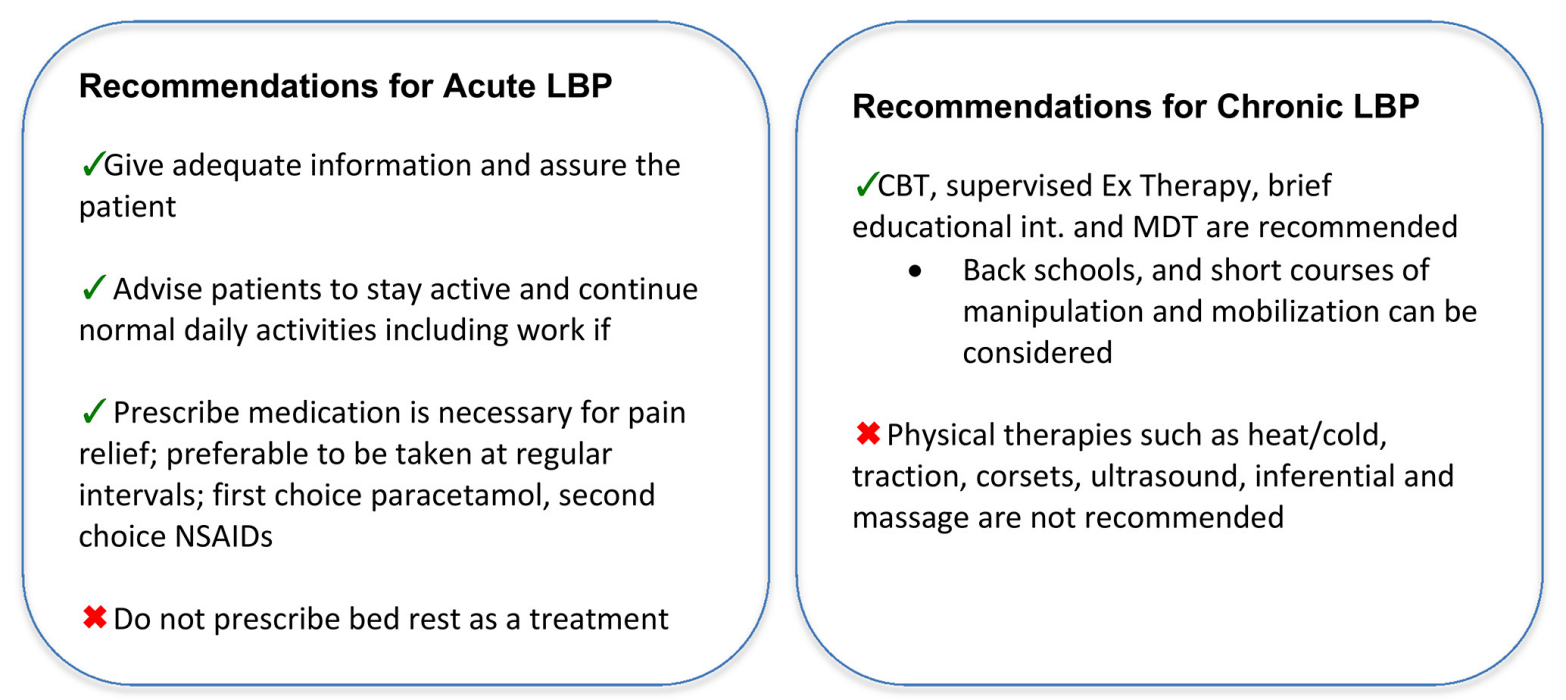
Summary of conservative treatment recommendations in the European LBP guidelines.

From the identified studies, original studies were included if they were a randomised controlled trial, included patients with non-specific low back pain of any duration, contained a cognitive behavioural intervention arm, contained a comparison arm of wait-list control/usual care (WL/UC), and/or guideline-based active treatment (GAT), and included one of the following outcomes: pain, disability, quality of life, or work disability. The European LBP guidelines for acute [23] and chronic [24] non-specific LBP were used to guide the identification of treatments for the GAT comparison (Fig 1). Full descriptions of the inclusion and exclusion criteria, including our intervention definition, are reported in Table 1.

**Table 1:** Inclusion / Exclusion Criteria and working definitions.

Variable	Description
Studies	• Study Design: Randomised controlled trial
Population	• RCTs were included if they assessed adult participants (males and females over the age of 18) with a clinical diagnosis of non-specific LBP +/- radiating leg pain (NICE, 2009a) of any duration.
	• Trials were excluded if they included participants with a pathological cause of LBP, such as: infection, neoplasm, metastasis, osteoporosis, rheumatoid arthritis, fractures, spinal canal stenosis, or nerve root compromise.
	•Participants with neurodegenerative conditions (such as, multiple sclerosis), or women experiencing LBP during pregnancy, were also excluded.
Intervention	• RCTs were included if they investigated a CB intervention for non-specific LBP.
	• As there is no consensus for a specific definition of CB interventions (Burton et al, 2005; Hansen et al, 2010), the review team developed a working definition to allow for transparency in selection of studies**:CB interventions were included if they met the following working definition *‘The intervention is explicitly or implicitly based on the CB model (where the use of CB in relation to the intervention is explicitly stated OR where the connection between thoughts*, *feelings and behaviours in relation to the intervention is implicitly described) AND it uses specific techniques to both change cognitions and change behaviours*.*’*
	• Psychological interventions that were not explicitly or implicitly based on the CB model were excluded. Interventions using techniques to change either cognitions (such as cognitive restructuring) or behaviours (such as operant conditioning), but not both, were also excluded.
	• CB interventions delivered by any health care professional were included, however, interventions delivered by lay personnel were excluded.
	• The delivery method was not restricted (e.g. delivery using face-to-face or with online methods were included).
	• In cases where treatments were multimodal, for example, including CB as a component of a comprehensive back school, the intervention was deemed eligible only when the main focus of the intervention was based on CB. For example, if an intervention consisted of six treatment sessions covering a wide range of components, and CB constituted only one of those sessions, it was not deemed eligible for inclusion as CB was not the main focus of the treatment.
Comparator(s)	• Two comparison arms were included:
	**(1) No treatment (WL/UC):** No treatment—A trial arm in which participants received no active treatment during the study period, this included studies with a wait-list (WL) comparison or a comparison defined as usual care (UC) in which no prescribed treatment was provided within the trial.
	**(2) Guideline-based Active Treatment (GAT):** A prescribed/supervised treatment in line with the European Guidelines (2009). A trial arm in which participants were allocated to receive an active treatment, in line with the European LBP guidelines, the details of which were specified in some way.
	• Studies comparing different types of CB intervention (e.g. one to one versus group interventions) were only included where a non-CB control arm was used as a comparison. Studies comparing CB interventions to a surgical comparator or other treatments not listed in the European Guidelines were be excluded.
	• Studies comparing a CB intervention to a drug based comparator were only to be included if the drug type and dosage were in line with the current European LBP guidelines (2009).
Outcomes	At least one measure of either; Pain, Disability, Quality of Life, Function, work-disability. If more than one measure was used to assess these variables, priority was assessed according to the following rules:
	• ***Pain*:** For pain, if more than one outcome measure is reported, the hierarchy will be VAS then NRS then single item measure, then multi-item measure. For quality of life, both the EQ-5D and the SF-36 or SF-12 are commonly used to assess general quality of life. If an included study reports more than one of these scales, they will be prioritized in the above order.
	• ***Condition-specific disability*:** For disability, if more than one outcome measure is reported, the Roland-Morris Questionnaire (RMQ) will be used in the analysis if available. Otherwise, the ODI, the QBPDS, and the PDI will be prioritised as ordered.
	• ***Quality of life*:** Both the EQ-5D and the SF-36 or SF-12 are commonly used to assess general quality of life. If an included study reports more than one of these scales, they will be prioritised in the above order. For the SF-12 and SF-36 quality of life measures, the summary scale (mental & physical health) will be prioritized. If the two components are only reported separately, the physical health component will be prioritized over the mental health component. If only the eight subscales are reported, the general health domain will be used in the analysis.
	• ***Work Disability*:** days off work is a commonly reported outcome. If an included study reports day off work, it may be eligible to be included in a meta-analyses if certain criteria are met between studies, i.e. comparable time-periods.
Language	No restrictions, translation where possible

** The working CB definition was determined by mapping and cross referencing the best available evidence pertaining to the definition of a CB intervention and included four sources: two expert discussion papers [25,26], a clinical competency tool for using CB interventions (CTS-R-Pain; [27]) and the DOH’s clinical competency criteria for delivering CB treatments for anxiety and depression [28].

#### Screening, data extraction and risk of bias assessment

* Screening and data extraction forms were piloted prior to study selection to ensure consistency among reviewers. All study titles and abstracts retrieved from the literature searches were combined in EndNote X10 and double screened by review authors (BC-100%, AH-50%, HR, 50%); subsequent full texts were also double screened. Double data extraction was inputted onto a standardised form, adapted from the Cochrane Back Review Group form, and included information on: patient characteristics (age, symptom duration and treatment allocation); intervention information (duration, dose, mode and provider), number and type of comparison groups; outcome information (measurement tool, assessment time point and response rate); and analysis information (numbers analysed, mean and standard deviation). Two reviewers assessed each study for risk of bias against the updated Cochrane CBRG criteria, which classifies risk of bias across 6 domains (selection bias, performance bias, detection bias, attritions bias, reporting bias and other bias) [29], and rated as “low”, “high” or “unclear” (handbook.cochrane.org, section 8.5d). With permission and in collaboration with colleagues who authored the most recent Cochrane Review on CBT for LBP [17], we used quality assessments from that review where these were available. All assessments were undertaken using the same tool, and by trained and experienced individuals. In situations where agreement was not achieved between the two assessors, a further review author (EW) was consulted. If either of the review authors were a (co-) author of one of the included studies, they were not involved in the assessment of that trial in this review.

#### Data cleaning

When available, multiple published sources were retrieved for each study to capture all study information. For clarification or further information, study authors were contacted. Where the standard deviation (SD) could not be obtained nor calculated from available data, imputation using the pooled SD from all the other studies in the same meta-analysis was planned [30]. Studies reporting only the median and range of outcomes were not included in the meta-analysis since it suggests that data was skewed [30]. For outcomes where data was not reported in a suitable format for a meta-analysis, a narrative summary was produced. Cluster RCTs were eligible for inclusion and where possible, effect measures and standard errors were extracted from an analysis which took clustering into account. If the reported results did not take clustering into account, we adjusted for this where possible by using the number of clusters and an estimate of the intracluster correlation coefficient [30].

Where a study contained a different number of eligible intervention and/or control groups, the eligible groups were pooled to create one effect size for the study to avoid double-counting and therefore biasing the meta-analyses [30].

### Data Synthesis

#### Meta-analyses

Heterogeneity was assessed from clinical, methodological and statistical perspectives. Statistical heterogeneity was assessed graphically with forest plots and statistically with the Chi-squared (χ^2^) test and the I^2^ statistic [31]. I^2^ statistics were interpreted as follows: 0% to 40% may not be important; 30% to 60% may represent moderate heterogeneity; 50 to 90% may represent substantial heterogeneity; 75% to 100% high heterogeneity [32]. Data was analysed using Stata IC 13.

Meta-analyses were performed using a random effects model [33]. The primary summary effect measure was the standardised mean difference (SMD) for all outcomes where data was measured with different instruments. Where applicable, scales were reversed by subtracting the mean score from the maximum score for that scale. A negative SMD indicated a treatment effect in favour of the CB intervention. Effect sizes proposed by Cohen [34] were used with 0.2 representing a small effect, 0.5 a moderate effect, and 0.8 a large effect.

#### Contrasts

Our primary contrast was the effect of CB versus GAT at long-term follow-up (closest to 52 weeks and >26weeks). We also included a short-term follow-up assessment (as close to 6 weeks but not exceeding 12 weeks) and a comparison to waitlist and usual care (WL/UC).

#### Reporting bias

Funnel plots were produced to assess for reporting bias. Asymmetry of funnel plots was assessed visually and using Egger’s test [35] when a minimum of 10 studies were included in the meta-analysis and the studies were not of similar size [36]. In the event of any detected asymmetry, sensitivity analyses were planned to consider the implications of bias on the meta-analysis.

#### Sub-group analyses

Based on previous evidence [37–39] we explored the treatment effect for studies that only included patients with acute (<6weeks) or persistent (>6 weeks) LBP through subgroup analyses. To explore baseline severity, studies were categorised according to the mean score for all participants at baseline on a pain scale and a back-specific disability measure; studies with a mean score of ≥60% of the scale maximum for both pain and disability were classified as high intensity [40] and analysed as separate subgroups.

We explored potential areas of heterogeneity by examining methodological quality, intervention and control features, and assessment time point variation. Risk of bias was based on five items likely to be associated with internal validity to calculate a summary score (allocation concealment, blinding of patients, blinding of outcome assessor, intention to treatment analysis, acceptable drop-out rate). Using the PRECIS tool [41], pragmatism was assessed on 3 items for both the intervention and control (i) training / expertise, (ii) protocol flexibility, and (iii) fidelity assessment. Therefore, studies were classified as (i) low risk of bias (having at 3 to 5 items) or high risk of bias (having 0–2 items), and (ii) high pragmatism (score of 3) or low pragmatism (score of 1–2). We planned to explore intervention and control intensity (total number of contact hours), however, we noted that as the intensity of the experimental intervention increased, so did the intensity of the control intervention. Thus, we chose not to explore this analysis since it would not have been possible to determine the extent intervention and control intensity influenced effect size.

#### Sensitivity analyses

We formally investigated influences on effect size at two levels: (i) methodological, (ii) concurrent treatments, and (iii) assessment time point variation. First, meta-analyses were repeated including only studies judged as low risk of bias (assessed as above). Secondly, additional sensitivity analyses were performed to examine the impact of concurrent treatments (those studies that evaluated the CB intervention in combination with the control intervention, such as, CB plus exercise vs exercise alone) on the summary effect size. Thirdly, meta-regression was used to assess the impact of assessment time point variation on the level of observed heterogeneity [42].

## Results

**Fig 2:**
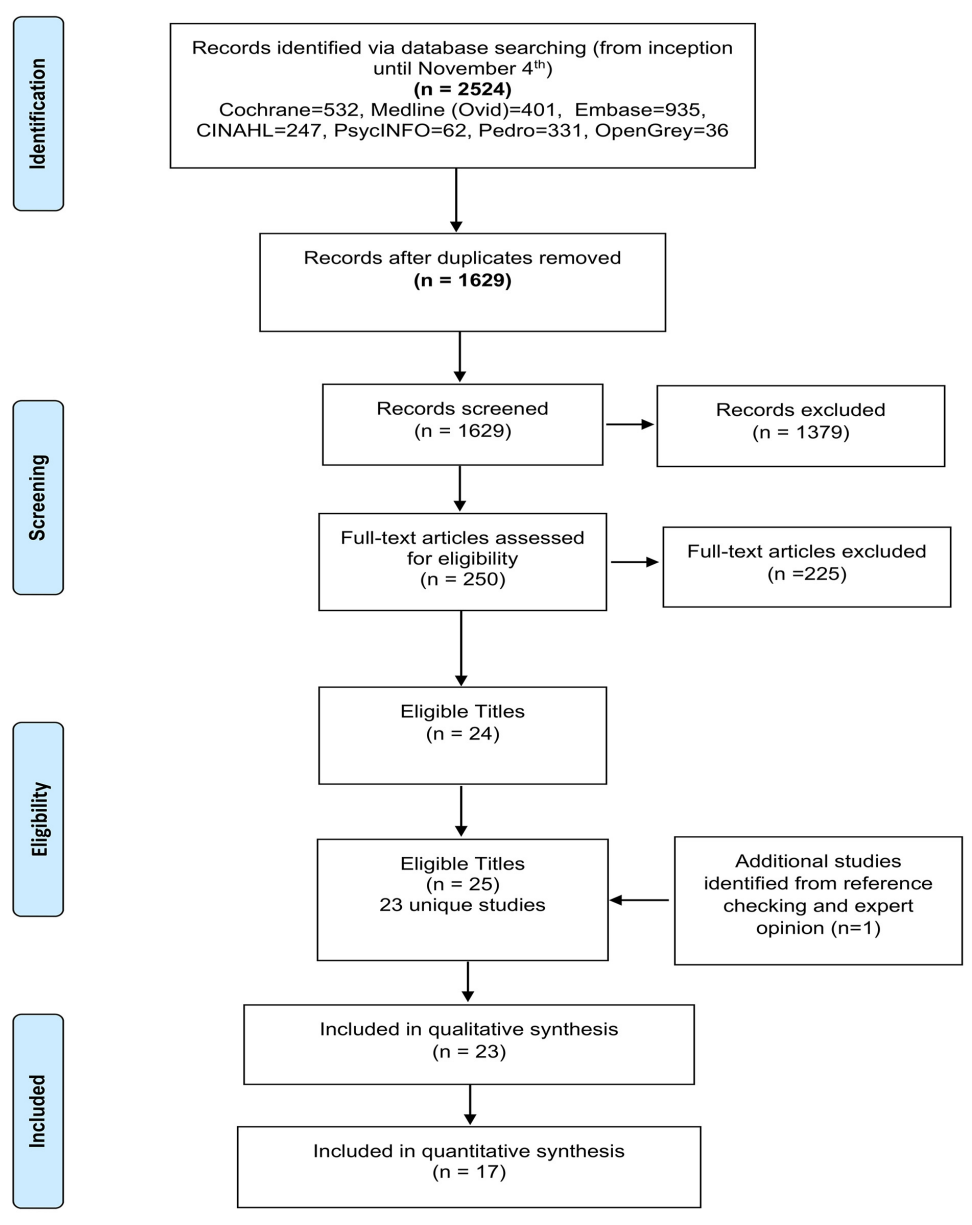
PRISMA Flow diagram.

We identified 1629 unique titles, from which 23 unique studies met the inclusion criteria (Fig 2). The 23 studies contained 3359 participants with non-specific LBP; with only 3 studies [22,43,44] including participants with pain of less than 6 weeks in duration (n = 373). CB interventions were delivered through three modes: group-based (n = 10), individual (n = 9), or combined (n = 4). Intervention duration varied between 1 to 52 weeks (average 8.4 weeks) and total contact time ranged from 20 minutes to 91 hours (average: 19 hours). Treatment providers included psychologists (n = 8), physiotherapists (n = 6), multiple professions (n = 5), GPs (n = 1), and self-directed (n = 3). Comparators included WL/UC (n = 10), GAT (n = 12), and both a WL/UC and a GAT comparison (n = 1). Pain and disability were the most frequently reported outcomes (ST: 87% and 61% and LT: 48% and 35% respectively). However, the choice of outcome measure, and the assessment time points, varied considerably between studies. A description of each study can be found in Tables 2 and 3.

**Table 2:** Description of study characteristics.

Study, Year	Pain (wks)	Age, M (SD)	Severity	CB* Mode (provider)	CB Duration (wks)	Contact time (hours)	Sample (n)
**Abbasi 2012**	≥ 6	45 (10)	Low	Group + Individual (Multiple HCPs*)	7	CB: 15	CB (22)
						WL/UC: 0	WL/UC (11)
**Altmaier 1992**	≥ 6	39.9 (8.91)	Low	Group + Individual (unclear)	3	CB: ~90	CB+C* (24)
						GAT: ~90	GAT (21)
**Basler 1997**	≥ 6	49.3 (9.7)	Low	Group (PSY*)	12	CB: 30	CB (36)
						WL/UC: 0	WL/UC (40)
**Buhrman 2004**	≥ 6	44.6 (10.4)	High	Individual (SD*+PSY-phone)	6	CB: 1	CB (22)
						WL/UC: 0	WL/UC (29)
**Buhrman 2011**	≥ 6	43.2 (9.8)	Low	Individual (SD+PSY-email)	8	CB: 1.5	CB (26)
						WL/UC: 0	WL/UC (28)
**Carpenter 2012**	≥ 6	42.5 (10.3)	Low	Individual (SD)	3	CB: 7.5	CB (70)
						WL/UC: 0	WL/UC (71)
**Christiansen 2010**	≥ 6	47.8 (9.4)	Low	Individual (PSY)	3	CB: 91	CB+C (34)
						GAT: 90	GAT (41)
**Critchley 2007**	≥ 6	44 (12.36)	Low	Group (PT*)	8	CB: 12	CB (69)
						GAT: ~9	GAT (41)
**Fersum 2013**	≥ 6	41.9 (11.36)	Low	Individual (PT)	12	CB: 5	CB (62)
						GAT: 4	GAT (59)
**Gohner 2006**	< 6	36.38 (11.85)	Low	Unclear (PSY)	6	CB: 6	CB+C (25)
						GAT:	GAT (22)
**Hill 2011**	≥ 6	49.8 (14.77)	High	Individual (PT)	12	CB 3.5	CB+C^#^ (157)
						GAT: 3	GAT (79)
Jellema 2005*	< 6	42.7 (11.6)		Individual (GP)	1	CB: 0.3	CB (143)
						WL/UC: 0	WL/UC (171)
**Johnson 2007**	≥ 6	47.9 (11.05)	Low	Group (PT)	6	CB: 16	CB (116)
						WL/UC	WL/UC (118)
**Johnstone 2004**	< 6	44.7 (13.2)	Low	Individual (PT)	6	CB: 4.5	CB (~6)
						GAT: 3	GAT (~6)
**Lamb 2010 / 2012**	≥ 6	53.3 (14.7)	Low	Group (PT)	7	CB: 10.5	CB+C (468)
						GAT: 0.25	GAT (233)
**Monticone 2013**	≥ 6	49.3 (7.5)	High	Individual (Multiple HCPs)	5	CB: 15	CB+C (45)
						GAT: 10	GAT (45)
**Moore 2000**	≥ 6	49.45 (10.6)	Low	Group + Individual (PSY)	4	CB: 4.8	CB (113)
						WL/UC: 0	WL/UC (113)
**Nicholas 1991**	≥ 6	41.2 (n/a)	Low	Group (PT+PSY)	5	CB: 17.5	CB+C (10)
						GAT: 17.5	*GAT(21)
**Nicholas 1992**	≥ 6	43.7 (n/a)	Low	Group (PT+PSY)	5	CB: 17.5	CB+C (10)
						GAT: 17.5	GAT(10)
**Schweikert 2006**	≥ 6	46.7 (9.1)	Low	Group + Individual (PSY)	3	CB: ~90	CB+C (200)
						GAT: ~90	GAT (209)
**Smeets 2006 / 2008**	≥ 6	41.9 (9.65)	Low	Group (PSY+SW*)	10	CB: 26.5	1. CB (60)
						CB+C: 79	2. CB+C (62)
						GAT: 52.5	3.CB±C (122)
							1. WL/UC (51)
							2. GAT (54)
							3. GAT (54)
**Turner 1988**	≥ 6	46 (n/a)	Low	Group (PSY)	8	CB: 16	CB (26)
						WL/UC: 0	WL/UC (25)
**Turner 1993**	≥ 6	42 (n/a)	Low	Group (PSY)	6	CB: 12	CB (25)
						WL/UC: 0	WL/UC (30)

CB–the CB group was a combination of two arms that contained a CB intervention. CB+C–a CB group in which the difference between the intervention and control groups was CB, so the CB group also received the control treatment. HCPs–Health Care professionals (e.g. physiotherapist, occupational therapists, nurses, psychologists) Jellema (2005)–this was a CRCT that was randomised at the GP practice level, however the unit of analysis was patient, and thus the sample size reported here is the number of patients. n/a–not applicable and described narratively in text. PSY–Psychologist, PT–Physiotherapist, SD–self-directed treatment with use of written handouts or online information packages, SW–Social Worker, wks–weeks.

^#^High risk group only

**Table 3:** Description of study outcomes.

Study, Year	Outcomes	Outcome tool used	FU (ST* or LT*)	FR*
**Abbasi 2012**	Pain	VAS*	7 wks—ST	89%
	Disability	RMDQ*	59 wks—LT	81%
**Altmaier 1992**	Pain	MPQ*	3 wks—ST	93%
	Disability	MPQ*		
	Work	n/a*		
**Basler 1997**	Pain	NRS*	12 wks—ST	81%
	Disability	DDS*		
	Work	n/a		
**Buhrman 2004**	Pain	MPQ*	6 wks—ST	91%
	Disability	MPQ*		
**Buhrman 2011**	Pain	MPQ	9 wks–ST	93%
	Disability	MPQ		
**Carpenter 2012**	RMDQ	RMDQ	3 wks—ST	93%
**Christiansen 2010**	Pain	NRS	3 wks—ST	80%
	Disability	Hanover*		
**Critchley 2007**	Disability	RMDQ	52 wks—LT	73%
	QoL	EQ-5D*		
	Work	n/a		
**Fersum 2013**	Pain	NRS	12 wks—ST	76%
	Disability	ODI*	64 wks—LT	72%
	Work	n/a		
**Gohner 2006**	Pain	NRS	6 wks—ST	94%
			33 wks—LT	87%
**Hill 2011** ^#^	Pain	unclear	52 wks—LT	78%
	Disability	RMDQ		
	QoL	SF-12*		
Jellema 2005*	Pain	0–10 NRS	6 wks—ST	97%
	Disability	RMDQ	52 wks—LT	92%
	QoL	SF-36 *		
	Work	n/a		
**Johnson 2007**	Pain	VAS	12 wks—ST	95%
	Disability	RMDQ	64 wks—LT	84%
	QoL	EQ-5D		
**Johnstone 2004**	Pain	VAS	~4 wks—ST	100%
	Disability	RMDQ		
**Lamb 2010 / 2012**	Pain	MVK*	12 wks—ST	78%
	Disability	RMDQ	52 wks–LT	85%
**Monticone 2013**	Pain	NRS	5 wks—ST	100%
	Disability	RMDQ	57 wks–LT	100%
	QoL	SF-36		
**Moore 2000**	Pain	NRS	12 wks–ST	94%
	Disability	RMDQ	52 wks—LT	85%
	QoL	SF-36		
**Nicholas 1991**	Pain	5pt likert	5 wks–ST	74%
			57 wks—LT	61%
**Nicholas 1992**	Pain	5pt likert	5 wks—ST	90%
**Schweikert 2006**	Back Pain	6pt likert	3 wks—ST	93%
	Disability	Hanover*		
	QoL	EQ-5D		
**Smeets 2006 / 2008**	Pain	VAS	10 wks—ST	1. 95%
	Disability	RMDQ		2. 92%
			62 wks—LT	3. 89%
**Turner 1988**	Pain	MPQ	8 wks—ST	88%
**Turner 1993**	Pain	VAS	6 wks—ST	71%

DDS–Dusseldorf Disability Scale, EQ-5D –European Quality of Life Scale, FR–response rate at follow-up assessment, Hanover–Hanover Functional Questionnaire, Jellema–this was a CRCT that was randomised at the GP practice level however, the unit of analysis was patient, thus, the sample size reported here is the number of patients, LT–Long term, MPQ–McGill Pain Questionnaire (pain intensity subscale for Pain outcome and pain interference subscale for disability outcome), MVK–Modified Von Korff, n/a–not applicable and described narratively in text, NRS– 11pt Numerical Rating Scale, ODI—Oswestry Disability Index, RMDQ–Roland Morris Disability Questionnaire, SF12 –General Health Short Form 12 item, SF-36 –General Health Short Form 36 item, ST–Short term, VAS–Visual Analog Scale, wks–weeks.

^#^High risk group only

### Sample size and methodological quality

**Fig 3:**
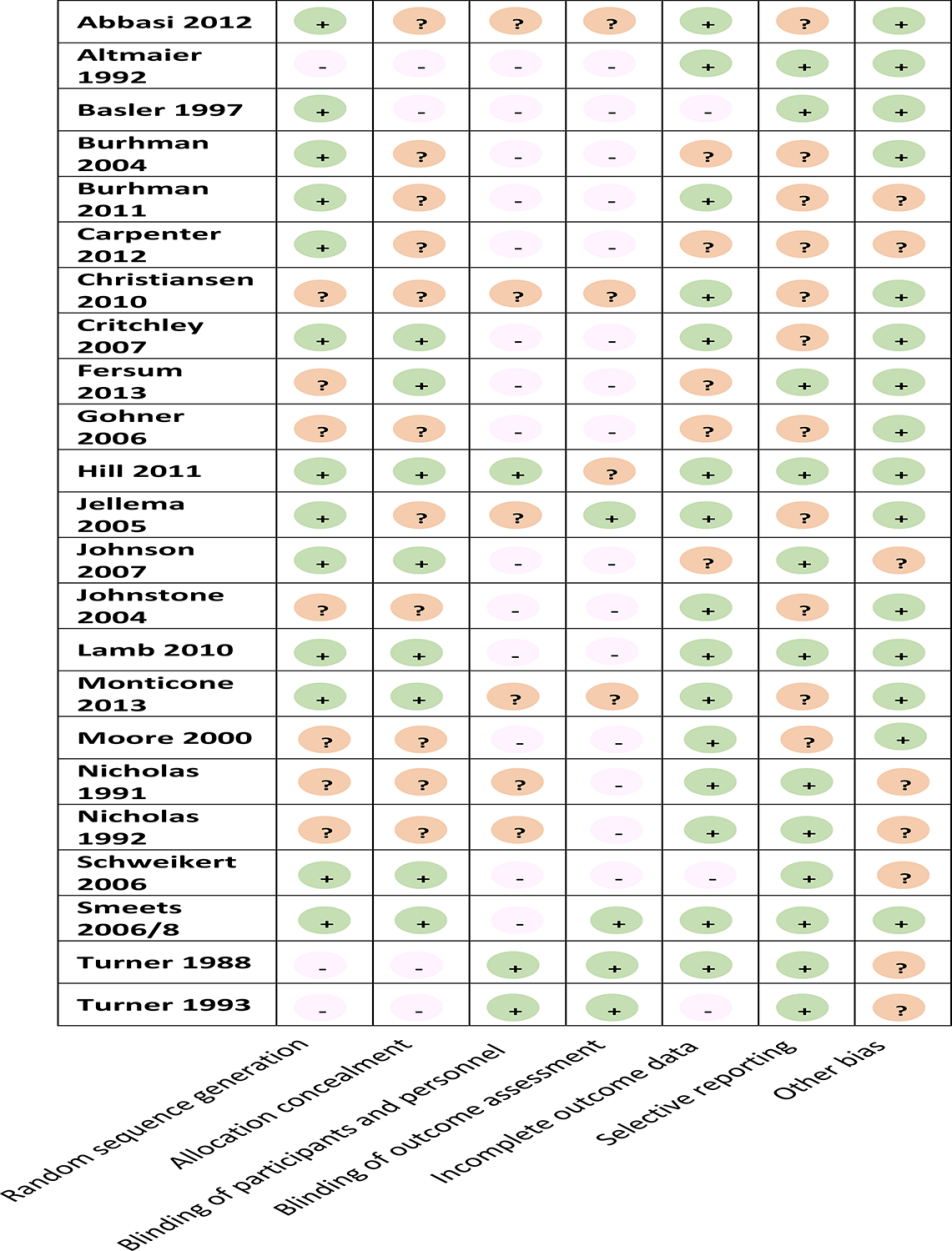
Risk of bias of included studies.

**Fig 4:**
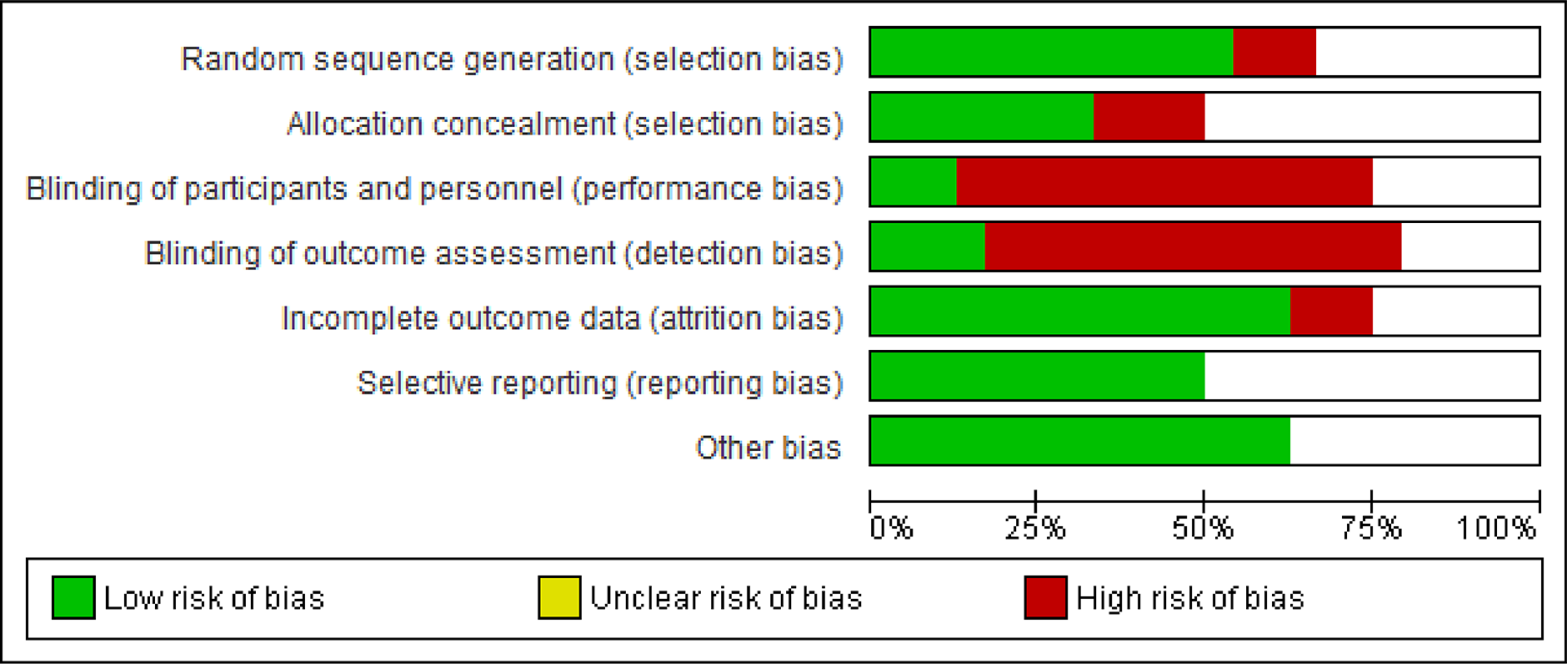
Summary of risk of bias for all studies.

Overall, sample sizes were moderate, though variable, ranging from 12 [22] to 701 [9] participants. Quality of reporting was poor and inconsistent leading to judgments of ‘unclear’ risk of bias in at least 25% of the six domains (Figs 3 and 4). While there was wide variation across studies on most items, over 80% of studies scored high or unclear on blinding of study participants and personnel, and blinding of outcome assessment.

### Meta analyses

**Fig 5:**
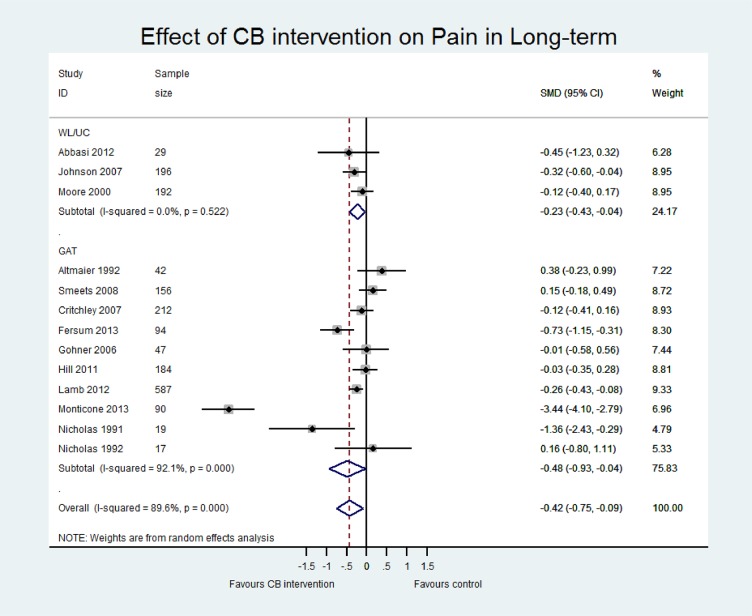
Forest plot of effect of CB on pain at long-term.

**Fig 6:**
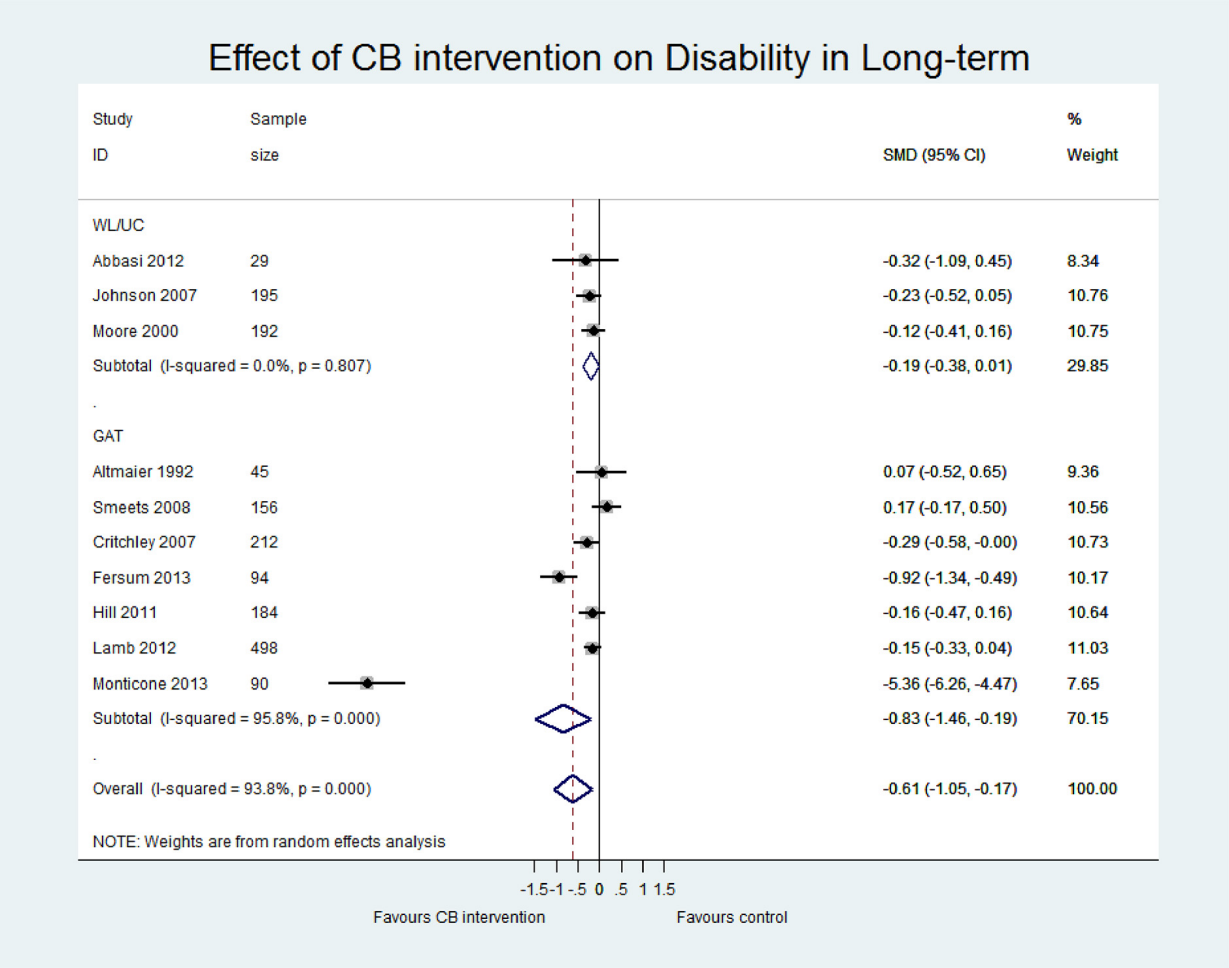
Forest plot of effect of CB on disability at long-term.

**Fig 7:**
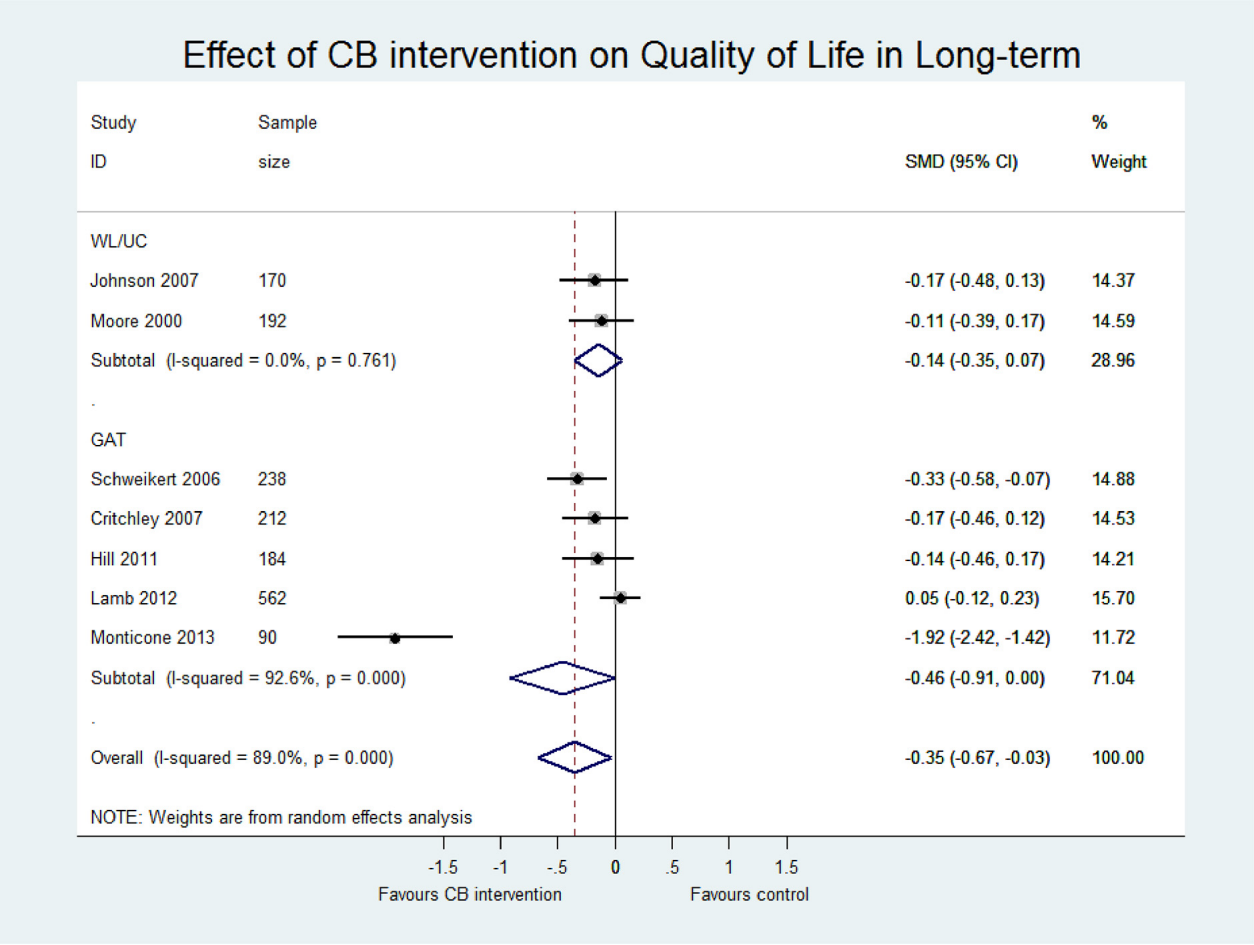
Forest plot of effect of CB on quality of life at long-term.

Table 4 presents an overview of the results including the standardised mean difference for both contrasts CB vs (i) WL/UC and (ii) GAT at both short term and long term for three outcomes (pain, disability and quality of life). Sensitivity analyses including only those studies with low risk of bias are also reported for each contrast. The main meta-analyses for pain, disability and quality of life outcomes at short and long term follow-up are shown in Figs 5–7 and in S2–S4 Figs.

**Table 4:** Summary of meta-analysis results.

		Pooled Effect Size (95% CI)							
		Short-term (All studies)	I^2^ (n)	Low RoB	I^2^ (n)	Long-term (All studies)	I^2^ (n)	Low RoB	I^2^ (n)
**Disability**	WL/UC	-0.25 (-0.46, -0.04)	53.6 (8)	-0.69 (-1.08, -0.29)	- (1)	-0.19 (-0.38, 0.01)	0.0 (3)	No suitable studies	- (0)
	GAT	-0.59 (-1.14, -0.03)	94.7 (7)	-0.12 (-0.29, 0.04)	0.0 (2)	-0.83 (-1.46, -0.19)	95.8 (7)	-0.13 (-0.29, 0.04)	30.1 (4)
	All	-0.38 (-0.66, -0.10)	89.1 (15)	-0.26 (-0.60, 0.08)	71.9 (3)	-0.61 (-1.05, -0.17)	93.8 (10)	-0.13 (-0.29, 0.04)	30.1 (4)
**Pain**	WL/UC	-0.31 (-0.48, -0.14)	25.6 (9)	-0.64 (-0.97, -0.31)	0.0 (2)	-0.23 (-0.43, -0.04)	0.0 (3)	No suitable studies	- (0)
	GAT	-0.46 (-0.84, -0.08)	89.0 (10)	-0.21 (-0.60, -0.08)	23.3 (2)	-0.48 (-0.93, -0.04)	92.1 (10)	-0.11 (-0.28, 0.07)	41.0 (4)
	All	-0.39 (-0.61, -0.18)	80.5 (19)	-0.34 (-0.60, -0.08)	53.8 (4)	-0.42 (-0.75, -0.09)	89.6 (13)	-0.11 (-0.28, 0.07)	41.0 (4)
**Quality of Life**	WL/UC	-0.18 (-0.38, 0.02)	0.0 (2)	No suitable studies	- (0)	-0.14 (-0.35, 0.07)	0.0 (2)	No suitable studies	- (0)
	GAT	-0.55 (-1.21, 0.10)	94.9 (3)	No suitable studies	- (0)	-0.46 (-0.91, 0.00)	92.6 (5)	-0.04 (-0.19, 0.11)	11.3 (3)
	All	-0.38 (-0.74, -0.01)	89.9 (5)	No suitable studies	- (0)	-0.35 (-0.67, -0.03)	89.0 (7)	-0.04 (-0.19, 0.11)	11.3 (3)

Effect sizes are all standardised mean differences. Negative effect sizes favour CB over control arm. d = 0.2 small, 0.5 moderate, 0.8 large. Where there was either no suitable studies or only 1 study included in the meta-analysis, there is no I^2^, thus a symbol of “-” has been inputted.

#### CB versus WL/UC

Pooled estimates at ST were small and statistically significant for pain (p<0.01, n = 9) and disability (p = 0.02, n = 8). Sensitivity analysis excluded many studies due to high risk of bias but did not show statistically different results. At long term, pooled estimates were still small and significant for pain (p = 0.02, n = 3) but did not reach significance for disability (p = 0.06; n = 3). No studies at long term were classified as low risk of bias and therefore sensitivity analysis was not performed. Only two studies reported quality of life data, which showed a small and insignificant effect in favour of CB at short and long term. Two studies reported work disability but the evidence remained inconclusive. One study had incomplete data, reporting the intervention group results only [45], and the other study found no significant between-group difference in work disability when assessed using a patient-reported binary (yes/no) measure of days lost at work [44].

#### CB vs GAT

Pooled effect estimates were moderate to large and statistically significant for pain and disability in both the short (pain; p = 0.02, n = 10; disability; p<0.01, n = 7) and long term (pain; p = 0.03, n = 10; disability; p = 0.01, n = 7). While effect sizes were moderate for quality of life, they were not statistically significant (LT p = 0.05, n = 5; ST p = 0.10, n = 3). However, we observed considerable heterogeneity in all comparisons (I^2^ >80%). There was a wide range in the magnitude of effects and while three studies showed large and significant effect sizes in favour of CB, the majority of studies showed small to moderate effect sizes that were insignificant at long term. Furthermore, a single study [46] with a particularly long intervention duration (52 weeks) may have influenced the pooled effect sizes due to its extremely large effects (SMD -5.36 for disability LT, compared to the second largest of SMD -0.92). While removing this study from the analyses considerably reduced the magnitude of the pooled SMDs, the estimates remained statistically significant and the heterogeneity for pain and disability outcomes remained substantial. Therefore, the pooled effect sizes should be viewed with caution and thus, we have presented a narrative synthesis of the studies to provide a more meaningful interpretation of the results.

#### Narrative synthesis

At long-term follow-up, 7 studies assessed disability, 10 assessed pain, and 5 assessed quality of life, with a wide range of reported effect sizes. The majority of studies reported effects in favour of CB, most of which were small to moderate and not statistically significant, with a smaller number reporting large and significant effect sizes. This wide range in effect sizes was also observed at short term. Due to the considerable statistical heterogeneity, we explored common factors that could explain the diversity in effect sizes including methodological design (risk of bias) and intervention and control characteristics (such as, pragmatism) (S1 Table. Information for GAT comparisons). Further details of the GAT treatments, such as dose and duration, are also reported in S1 Table. Information for GAT comparisons.

Restricting on methodological quality reduced heterogeneity to a moderate level at short and long term for all outcomes. In the subgroup of low risk of bias studies (n = 4), effect sizes remained in favour of CB, were smaller and more precise, and were either approaching significance (n = 4) or significant (n = 1). Subgrouping according to the PRECIS tool classification did not reduce heterogeneity or influence the pooled effect estimates.

#### Pre-planned subgroup and sensitivity analyses

Analysis by pain duration was not performed since only 1 study had a duration of <6 weeks. In terms of severity, 3 studies were classified as having high severity on pain and disability at baseline [10,46,47]. There were no significant differences in the effect sizes between these subgroups. Sensitivity analyses including only concurrent treatments (CB + GAT vs GAT, n = 10) had minimal impact on the pooled effect sizes. Results from the meta-regression indicated that the time point of assessment did not explain the high levels of heterogeneity for pain and disability at both short and long term time points.

## Discussion

### Summary

To our knowledge this is the first systematic review that has investigated the effects of CB interventions for patients with non-specific LBP of any duration and of any age, aiming to reflect the clinical population. The review included 23 studies with a total of 3359 participants and pooled effect estimates suggest small to moderate effect sizes in favour of CB interventions on a range of patient reported outcomes when compared to no treatment arm or a guideline-based active treatment. This review provides evidence that CB interventions are clinically effective and worthwhile for non-specific LBP, and this appears robust across a range of presentations and sample characteristics. These effects appear to be maintained over time, with patients followed up for an average of 54 weeks for disability and 49 weeks for pain.

### Comparison with other studies

Firstly, our results comparing CB interventions to wait-list/usual care are consistent with the findings of previous systematic reviews and meta analyses of CB for LBP [16,17], which reported moderate effects in favour of CB on pain and disability in the short term. Thus, the results of this review and previous reviews show that compared to wait-list or usual care, CB appears to have a beneficial effect.

Our review is the first to compare CB treatments to other guideline-based active treatments solely for a LBP population. While we acknowledge the heterogeneity within this comparison, visual inspection indicates that for the majority of studies, when compared to other typical physiotherapy-based treatments, a CB intervention is more effective. When a single study with large effect sizes was removed from our analyses, the pooled estimates were reduced and were more consistent with previous meta-analyses of CB versus other active treatments for patients with non-malignant pain [48]. Thus, it is likely that the true effect of CB versus other guideline based active treatments may range from small to moderate. When interpreting the clinical significance of these effect sizes, most studies maintained a 30% decrease on the RMDQ at long term, which is considered clinically meaningful [49]. It is also worth noting that the included trials varied in their degree of pragmatism. Since we expect effect sizes found in more pragmatic trials to be smaller for a given amount of clinical change, these small effect sizes may reflect clinically important changes [50].

### Strengths and limitations

Our review used a rigorous approach in line with the Cochrane guidelines which included a sensitive search strategy in multiple databases (including grey literature), and ensured that all study processes (screening, data checking, and risk of bias assessment) were completed by two authors. In line with PRISMA guidelines, we assessed the level of reporting bias and influence of methodological quality. By not limiting our patient inclusion criteria by duration of pain or age, we were able to include more participants, making our results more precise and applicable to the typical clinical population of LBP patients. Additionally, we selected contrasts that would be meaningful for health care professionals and policy makers, excluding studies with active treatment comparators not recommended in the European LBP guidelines. Moreover, since there is no consensus on the definition of CB treatment for LBP in the literature, we used clear and transparent criteria to assess the eligibility of study interventions.

Limitations included the exclusion of a small number of studies from the meta-analysis because of poor reporting of study data. The search term used for interventions was ‘cognitive-behavioural’, and hence where investigators have tested a CB intervention and not identified it as such, we would not have identified these papers. Lastly, considerable heterogeneity was observed on all outcomes when CB was compared to GAT (I^2^ > 80%). Heterogeneity was partially explained by methodological quality. Other reasons could include differences in the interventions and lack of consistency in the reference (control) treatments. Despite exploring various factors, such as intervention and control characteristics, we found no single factor that could explain the heterogeneity amongst studies.

### Clinical Implications

Nearly all included studies found clinically meaningful effects in favour of the CB intervention, with CB outperforming the majority of GAT comparisons. GAT treatments encompassed typical physiotherapy management and included a mixture of education, home and clinical based exercise, and some passive modalities (including manual therapy), indicating that for the most part, the management of patients with LBP can be improved by using a CB intervention.

Our results suggest that CB interventions can be successfully delivered by a range of health professionals. However, on the whole, interventions were poorly reported, hindering implementation in practice. To this end, we recommend that future studies use the TiDier guidelines to describe the intervention [51].

### Future Work

The individual effect sizes varied markedly in magnitude and, on closer inspection, it was clear that individual study CB interventions also varied considerably on key factors, such as intervention content, dose, method of delivery, and provider. Whilst further research on these differences in these factors may be of interest, there were several examples of lower intensity, low cost interventions that were effective. Thus, future work should focus on integrating these interventions into clinical practice.

## Conclusions

In conclusion, our results suggest that CB interventions have a long-term beneficial effect on pain, disability and quality of life in comparison to no treatment and other guideline-based active treatments for patients with LBP of any duration and of any age.

## Supporting Information

S1 Dataset Outcome data for disability. (PDF)

S2 Dataset Outcome data for pain. (PDF)

S3 Dataset Outcome data for quality of life. (PDF)

S1 Fig Search strategy. (DOCX)

S2 Fig Forest plot of effect of CB on pain at short-term. (TIF)

S3 Fig Forest plot of effect of CB on disability at short-term. (TIF)

S4 Fig Forest plot of effect of CB on quality of life at short-term. (TIF)

S1 Table Information for GAT comparisons. (DOCX)

S2 Table PRISMA 2009 checklist. (DOC)

S1 Text Registered protocol (PROSPERO). (PDF)
