# Supplementary material for: The Effectiveness of Cognitive Behavioural Treatment for Non-Specific Low Back Pain: A Systematic Review and Meta-Analysis
Source: PLoS One. 2015 Aug 5;10(8):e0134192. doi: 10.1371/journal.pone.0134192 (PMC4526658; doi:10.1371/journal.pone.0134192)
Supplement: S1 Dataset — (PDF) [file pone.0134192.s001.pdf]

trialid	riskofbias	LBP_duration	assessmentpoint_weeks	timeperiod	cb_code	cb_mean	cb_sd	cb_n
Abbasi 2012	High risk of bias	>=6 weeks	59 weeks	LT	CB alone	8.515789474	5.6702	19
Abbasi 2012	High risk of bias	>=6 weeks	7 weeks	ST	CB alone	6.028571429	3.8727	21
Basler 1997	High risk of bias	>=6 weeks	12 weeks	ST	CB alone	1.63	0.87	36
Buhrman 2004	High risk of bias	>=6 weeks	6 weeks	ST	CB alone	3.2	1.4	22
Buhrman 2011	High risk of bias	>=6 weeks	12 weeks?? 9 weeks	ST	CB alone	3.2	1.4	23
Carpenter 2012	High risk of bias	>=6 weeks	3 weeks (end of program)	ST	CB alone	13.5	5.8	63
Johnson 2007	High risk of bias	>=6 weeks	65 weeks	LT	CB alone	6.7	5.6	101
Johnson 2007	High risk of bias	>=6 weeks	12 weeks	ST	CB alone	7.4	5.3	110
Moore 2000	High risk of bias	>=6 weeks	52 weeks	LT	CB alone	4.84	6.01	97
Moore 2000	High risk of bias	>=6 weeks	12 weeks	ST	CB alone	5.39	5.76	108
Smeets 2006	Low risk of bias	>=6 weeks	10 weeks	ST	CB alone	-2.65	4.66	55
Altmaier 1992	High risk of bias	>=6 weeks	3 weeks	ST	CB + Control	57.43	15.06	24
Altmaier 1992	High risk of bias	>=6 weeks	29 weeks	LT	CB + Control	52.19	19.58	24
Christiansen 2010	High risk of bias	>=6 weeks	3 weeks	ST	CB + Control	69.6	13.4	30
Schweikert 2006	High risk of bias	>=6 weeks	3 weeks (discharge)	ST	CB + Control	2.8	12.3	169
Smeets 2006	Low risk of bias	>=6 weeks	10 weeks	ST	CB + Control	-2.27	4.19	55
Smeets 2008	Low risk of bias	>=6 weeks	62 weeks	LT	CB + Control	-2.438285714	4.442429909	105
Critchley 2007	Low risk of bias	>=6 weeks	52 weeks	LT	CB alone	5.8	6.780917439	69
Fersum 2013	High risk of bias	>=6 weeks	12 weeks	ST	CB alone	7.6	6.7	51
Fersum 2013	High risk of bias	>=6 weeks	64 weeks	LT	CB alone	9.9	9.8	51
Hill 2011	Low risk of bias	>=6 weeks	52 weeks	LT	CB + Control	5.9	7.2	128
Lamb 2012	Low risk of bias	>=6 weeks	12 weeks	ST	CB + Control	6.5	5.27	335
Lamb 2012	Low risk of bias	>=6 weeks	52 weeks	LT	CB + Control	6.1	5.62	339
Monticone 2013	High risk of bias	>=6 weeks	5 weeks	ST	CB + Control	5.04	2.04	45
Monticone 2013	High risk of bias	>=6 weeks	57 weeks	LT	CB + Control	1.31	1.59	45

control_code	control_mean	control_sd	control_n	outcome	measure	measuremax
WL/UC	10.4	6.2	10	disability	RDQ	24
WL/UC	3.2	3.2	11	disability	RDQ	24
WL/UC	1.84	0.62	40	disability	Dusselsorf Disability Scale - physical functions subscale	5
WL/UC	3.5	1.2	29	disability	MPI - interference subscale	6
WL/UC	3.5	1.2	27	disability	MPI - interference subscale	6
WL/UC	16.3	5.2	68	disability	RMDQ	24
WL/UC	8	5.5	94	disability	RMDQ	24
WL/UC	8	5.3	113	disability	RMDQ	24
WL/UC	5.56	5.8	95	disability	RMDQ	24
WL/UC	6.55	6.15	105	disability	RMDQ	24
WL/UC	0.04	2.9	50	disability	RMDQ	24
GAT	57.67	16.37	21	disability	MPI - interference subscale	30
GAT	50.71	25.95	21	disability	MPI - interference subscale	30
GAT	70.6	17.1	30	disability	HannoverADL	100
GAT	3.5	13.4	194	disability	Hanover Functional questionnaire	100
GAT	-2.25	4.51	52	disability	RMDQ	24
GAT	-3.2	4.81	51	disability	RMDQ	24
GAT	7.848251748	7.115221223	143	disability	RMDQ	24
GAT	18.5	8.1	43	disability	ODI	100
GAT	19.7	11.7	43	disability	ODI	100
GAT	4.8	6.3	56	disability	RMDQ	24
GAT	7.3	5.28	179	disability	RDQ	24
GAT	6.9	5.12	159	disability	RDQ	24
GAT	11.04	2.27	45	disability	RMDQ	24
GAT	11	2	45	disability	RMDQ	24

higherisgood	cb_meanadj	control_meanadj	control_class	samplesize	SMD_disability_ST	seSMD_disability_ST	SMD_disability_LT
No	8.515789032	10.39999962	UC	29			-0.321969956
No	6.028571606	3.200000048	UC	32	0.772364855	0.385318816	
No	1.629999995	1.840000033	UC	76	-0.280478537	0.230888113	
No	3.200000048	3.5	WL	51	-0.23264499	0.283706874	
No	3.200000048	3.5	WL	50	-0.231569886	0.284734756	
No	13.5	16.29999924	WL	131	-0.509410024	0.177720875	
No	6.699999809	8	UC	195			-0.234148279
No	7.400000095	8	UC	223	-0.113207527	0.13405022	
No	4.840000153	5.559999943	UC	192			-0.121888459
No	5.389999866	6.550000191	UC	213	-0.194780603	0.137378946	
No	-2.650000095	0.039999999	WL	105	-0.685792804	0.201158851	
No	57.43000031	57.66999817	GAT	45	-0.015303137	0.298811704	
No	52.18999863	50.70999908	GAT	45			0.065010361
Yes	30.39999962	29.39999962	GAT	60	0.065096527	0.258269608	
Yes	97.19999695	96.5	GAT	363	0.054264326	0.105241992	
No	-2.269999981	-2.25	GAT	107	-0.004599419	0.193423599	
No	-2.438285828	-3.200000048	GAT	156			0.166859016
No	5.800000191	7.84825182	GAT	212			-0.292243481
No	7.599999905	18.5	GAT	94	-1.478530169	0.233975157	
No	9.899999619	19.70000076	GAT	94			-0.915093362
Yes	18.10000038	19.20000076	GAT	184			-0.158493772
No	6.5	7.300000191	GAT	514	-0.151702553	0.092704594	
No	6.099999905	6.900000095	GAT	498			-0.14636758
No	5.039999962	11.03999996	GAT	90	-2.780266762	0.297261238	
No	1.309999943	11	GAT	90			-5.363463879

seSMD_disability_LT	_ES	_seES	_LCI	_UCI	_WT
0.393130124	-0.321969956	0.393130124	-1.092490792	0.44855094	8.337352753
					0
					0
					0
					0
					0
0.143810019	-0.234148279	0.143810019	-0.516010761	0.047714192	10.75773335
					0
0.144480765	-0.121888459	0.144480765	-0.405065536	0.161288634	10.75322437
					0
					0
					0
0.298889369	0.065010361	0.298889369	-0.520802021	0.650822759	9.36367321
					0
					0
					0
0.170944571	0.166859016	0.170944571	-0.168186188	0.501904249	10.5621109
0.147272229	-0.292243481	0.147272229	-0.580891728	-0.003595213	10.73427391
					0
0.217749223	-0.915093362	0.217749223	-1.341874003	-0.488312751	10.16824818
0.160432711	-0.158493772	0.160432711	-0.472936094	0.155948564	10.64103127
					0
0.096232824	-0.14636758	0.096232824	-0.334980458	0.042245295	11.03092766
					0
0.455951601	-5.363463879	0.455951601	-6.25711298	-4.469815254	7.651422977
