# Supplementary material for: The Effectiveness of Cognitive Behavioural Treatment for Non-Specific Low Back Pain: A Systematic Review and Meta-Analysis
Source: PLoS One. 2015 Aug 5;10(8):e0134192. doi: 10.1371/journal.pone.0134192 (PMC4526658; doi:10.1371/journal.pone.0134192)
Supplement: S2 Dataset — (PDF) [file pone.0134192.s002.pdf]

trialid	riskofbias	LBP_duration	assessmentpoint_weeks	timeperiod	CBcode	cb_mean	cb_sd	cb_n	controlcode
Abbasi 2012	High risk of bias	>=6 weeks	59 weeks	LT	CB alone	3.273684211	2.596037705	19	WL/UC
Abbasi 2012	High risk of bias	>=6 weeks	7 weeks	ST	CB alone	2.771428571	1.918332609	21	WL/UC
Basler 1997	High risk of bias	>=6 weeks	12 weeks	ST	CB alone	4.08	2.11	36	WL/UC
Buhrman 2004	High risk of bias	>=6 weeks	6 weeks	ST	CB alone	2.4	1.1	22	WL/UC
Buhrman 2011	High risk of bias	>=6 weeks	9 weeks	ST	CB alone	3.15	2.2	23	WL/UC
Johnson 2007	High risk of bias	>=6 weeks	12 weeks	ST	CB alone	29.1	24.5	110	WL/UC
Johnson 2007	High risk of bias	>=6 weeks	65 weeks	LT	CB alone	27.9	26.1	102	WL/UC
Moore 2000	High risk of bias	>=6 weeks	52 weeks	LT	CB alone	2.74	2.09	97	WL/UC
Moore 2000	High risk of bias	>=6 weeks	12 weeks	ST	CB alone	3.69	2.05	108	WL/UC
Smeets 2006	Low risk of bias	>=6 weeks	10 weeks	ST	CB alone	38.59	19.69449983	52	WL/UC
Turner 1988	Low risk of bias	>=6 weeks	8 weeks	ST	CB alone	15.91	11.63	24	WL/UC
Turner 1993	High risk of bias	>=6 weeks	6 weeks	ST	CB alone	44.3	28.45	21	WL/UC
Altmaier 1992	High risk of bias	>=6 weeks	29 weeks	LT	CB + Control	2.33	0.8	21	GAT
Altmaier 1992	High risk of bias	>=6 weeks	3 weeks	ST	CB + Control	2.05	0.74	21	GAT
Christiansen 2010	High risk of bias	>=6 weeks	3 weeks	ST	CB + Control	3.7	2.1	30	GAT
Schweikert 2006	High risk of bias	>=6 weeks	3 weeks	ST	CB + Control	-1.2	1.2	170	GAT
Smeets 2006	Low risk of bias	>=6 weeks	10 weeks	ST	CB + Control	44.67	19.25086574	51	GAT
Smeets 2008	Low risk of bias	>=6 weeks	62 weeks	LT	CB + Control	-1.332285714	24.17674862	105	GAT
Critchley 2007	Low risk of bias	>=6 weeks	52 weeks	LT	CB alone	38	36.02	69	GAT
Fersum 2013	High risk of bias	>=6 weeks	64 weeks	LT	CB alone	2.3	2	51	GAT
Fersum 2013	High risk of bias	>=6 weeks	12 weeks	ST	CB alone	1.7	1.7	51	GAT
Gohner 2006	High risk of bias	<6 weeks	33 weeks	LT	CB + Control	1.96	1.4	25	GAT
Gohner 2006	High risk of bias	<6 weeks	7 weeks	ST	CB + Control	3.04	1.7	26	GAT
Hill 2011	Low risk of bias	>=6 weeks	52 weeks	LT	CB + Control	3.7	2.7	128	GAT
Lamb 2012	Low risk of bias	>=6 weeks	12 weeks	ST	CB + Control	46.76	23.13	354	GAT
Lamb 2012	Low risk of bias	>=6 weeks	52 weeks	LT	CB + Control	44.5	26.04	392	GAT
Monticone 2013	High risk of bias	>=6 weeks	57 weeks	LT	CB + Control	1.38	1.07	45	GAT
Monticone 2013	High risk of bias	>=6 weeks	5 weeks	ST	CB + Control	2.69	0.97	45	GAT
Nicholas 1991	High risk of bias	>=6 weeks	57 weeks	LT	CB + Control	1.88	0.65	6	GAT
Nicholas 1991	High risk of bias	>=6 weeks	5 weeks	ST	CB + Control	1.81	0.8	7	GAT
Nicholas 1992	High risk of bias	>=6 weeks	31 weeks	LT	CB + Control	2.89	0.64	9	GAT
Nicholas 1992	High risk of bias	>=6 weeks	5 weeks	ST	CB + Control	3.07	0.79	9	GAT

control_mean	control_sd	control_n	measure	measuremax	higherisgood	cb_meanadj
4.3	1.4	10	0-10 VAS	10	No	3.273684263
3.2	1.6	11	0-10 VAS	10	No	2.771428585
4.18	1.37	40	0-10 NRS	10	No	4.079999924
3.2	0.8	29	Multidimesional Pain Inventory - pain severity subscale	6	No	2.400000095
3.35	2.6	27	Multidimesional Pain Inventory - pain severity subscale	6	No	3.150000095
35.3	26.7	113	100mm VAS	100	No	29.10000038
36.4	27.3	94	100mm VAS	100	No	27.89999962
2.98	1.99	95	0-10	10	No	2.74000001
4.06	2.17	105	0-10	10	No	3.690000057
53.35	22.6	50	VAS	100	No	38.59000015
22.14	12.35	21	The McGill Pain Questionniare Pain Rating Index		No	15.90999985
48.06	20.97	18	VAS	100	No	44.29999924
2	0.95	21	McGill Question	5	No	2.329999924
2	0.89	21	McGill Question	5	No	2.049999952
4.2	2.2	30	NRS	10	No	3.700000048
-1.2	1.2	193	6pt likert sale	6	No	-1.200000048
45.12	19.81529605	56	VAS	100	No	44.66999817
2.31	24.15697474	51	VAS	100	Yes	101.332283
42	30.2	143	0-100	100	No	38
3.8	2.1	43	0-10 NRS	10	No	2.299999952
3.8	1.9	43	0-10 NRS	10	No	1.700000048
1.98	1.98	22	0-10 NRS	10	No	1.960000038
2.91	2.02	25	0-10 NRS	10	No	3.039999962
3.6	3.2	56	Unclear	10	Yes	6.300000191
52.9	22.74	189	Modified Von Korff	100	No	46.75999832
51	23.93	195	Modified Von Korff	100	No	44.5
5.33	1.22	45	NRS	10	No	1.379999995
4.96	1.27	45	NRS	10	No	2.690000057
2.9	0.788761976	13	0-5	5	No	1.879999995
3.086875	0.712821356	16	0-5	5	No	1.809999943
2.75	1.11	8	0-5	5	No	2.890000105
2.72	0.77	9	0-5	5	No	3.069999933

control_meanadj	samplesize	STsmd	STse	LTsmd	LTse	SMD_pain_ST	seSMD_pain_ST	SMD_pain_LT	seSMD_pain_LT
4.300000191	29			-0.452412486	0.39550209			-0.452412486	0.39550209
3.200000048	32	-0.235682875	0.373435289			-0.235682875	0.373435289		
4.179999828	76	-0.056842905	0.229781657			-0.056842905	0.229781657		
3.200000048	51	-0.850733757	0.295503795			-0.850733757	0.295503795		
3.349999905	50	-0.082478531	0.283877015			-0.082478531	0.283877015		
35.29999924	223	-0.241823435	0.134435013			-0.241823435	0.134435013		
36.40000153	196			-0.318566978	0.143888071			-0.318566978	0.143888071
2.980000019	192			-0.117581427	0.144471377			-0.117581427	0.144471377
4.059999943	213	-0.17535539	0.137316912			-0.17535539	0.137316912		
53.34999847	102	-0.697276056	0.204112068			-0.697276056	0.204112068		
22.13999939	45	-0.520455956	0.304031938			-0.520455956	0.304031938		
48.06000137	39	-0.148678482	0.321672708			-0.148678482	0.321672708		
2	42			0.375764936	0.311453193			0.375764936	0.311453193
2	42	0.061091501	0.308682263			0.061091501	0.308682263		
4.199999809	60	-0.232495174	0.259099692			-0.232495174	0.259099692		
-1.200000048	363	0	0.105184123			0	0.105184123		
45.11999893	107	-0.023019647	0.193565264			-0.023019647	0.193565264		
97.69000244	156			0.15069221	0.170895815			0.15069221	0.170895815
42	212			-0.124223821	0.146705493			-0.124223821	0.146705493
3.799999952	94			-0.73304522	0.213972121			-0.73304522	0.213972121
3.799999952	94	-1.170521259	0.22429882			-1.170521259	0.22429882		
1.980000019	47			-0.011795647	0.292328745			-0.011795647	0.292328745
2.910000086	51	0.06975691	0.280198485			0.06975691	0.280198485		
6.400000095	184			-0.034960978	0.160227969			-0.034960978	0.160227969
52.90000153	543	-0.267012119	0.090453058			-0.267012119	0.090453058		
51	587			-0.256311804	0.087950982			-0.256311804	0.087950982
5.329999924	90			-3.442404509	0.33432743			-3.442404509	0.33432743
4.960000038	90	-2.008849621	0.259563655			-2.008849621	0.259563655		
2.900000095	19			-1.358881831	0.545802474			-1.358881831	0.545802474
3.086874962	23	-1.728356242	0.525814891			-1.728356242	0.525814891		
2.75	17			0.157171264	0.486759216			0.157171264	0.486759216
2.720000029	18	0.448680967	0.478030652			0.448680967	0.478030652		

_ES	_seES	_LCI	_UCI	_WT
-0.452412486	0.39550209	-1.227582335	0.322757334	6.276983738
				0
				0
				0
				0
				0
-0.318566978	0.143888071	-0.600582421	-0.036551528	8.95059967
-0.117581427	0.144471377	-0.400740117	0.165577248	8.945877075
				0
				0
				0
				0
0.375764936	0.311453193	-0.234672084	0.986201942	7.221377373
				0
				0
				0
				0
0.15069221	0.170895815	-0.184257433	0.485641867	8.717989922
-0.124223821	0.146705493	-0.411761314	0.163313672	8.927659988
-0.73304522	0.213972121	-1.152422905	-0.313667566	8.297485352
				0
-0.011795647	0.292328745	-0.58474946	0.56115818	7.438874722
				0
-0.034960978	0.160227969	-0.349002004	0.279080063	8.813154221
				0
-0.256311804	0.087950982	-0.428692579	-0.083931044	9.330347061
-3.442404509	0.33432743	-4.09767437	-2.787134886	6.961012363
				0
-1.358881831	0.545802474	-2.42863512	-0.289128661	4.786525726
				0
0.157171264	0.486759216	-0.796859264	1.111201763	5.332112789
				0
