# Supplementary material for: The Effectiveness of Cognitive Behavioural Treatment for Non-Specific Low Back Pain: A Systematic Review and Meta-Analysis
Source: PLoS One. 2015 Aug 5;10(8):e0134192. doi: 10.1371/journal.pone.0134192 (PMC4526658; doi:10.1371/journal.pone.0134192)
Supplement: S3 Dataset — (PDF) [file pone.0134192.s003.pdf]

trialid	riskofbias	LBP_duration	assessmentpoint_weeks	timeperiod	cb_code	cb_mean	cb_sd	cb_n	control_code
Johnson 2007	High risk of bias	>=6 weeks	65 weeks	LT	CB alone	0.75	0.23	89	WL/UC
Johnson 2007	High risk of bias	>=6 weeks	12 weeks	ST	CB alone	0.75	0.18	98	WL/UC
Moore 2000	High risk of bias	>=6 weeks	12 weeks	ST	CB alone	75.6	17.9	108	WL/UC
Moore 2000	High risk of bias	>=6 weeks	52 weeks	LT	CB alone	75.5	18	97	WL/UC
Schweikert 2006	High risk of bias	>=6 weeks	29 weeks	LT	CB + Control	70	17.7	105	GAT
Schweikert 2006	High risk of bias	>=6 weeks	3 weeks	ST	CB + Control	70.3	19.3	158	GAT
Critchley 2007	Low risk of bias	>=6 weeks	52 weeks	LT	CB alone	0.72	0.381426606	69	GAT
Hill 2011	Low risk of bias	>=6 weeks	52 weeks	LT	CB + Control	-8.6	12.2	128	GAT
Lamb 2012	Low risk of bias	>=6 weeks	52 weeks	LT	CB + Control	46.4	11.51	375	GAT
Lamb 2012	Low risk of bias	>=6 weeks	12 weeks	ST	CB + Control	47	11.44	332	GAT
Monticone 2013	High risk of bias	>=6 weeks	57 weeks	LT	CB + Control	85	13.81	45	GAT
Monticone 2013	High risk of bias	>=6 weeks	5 weeks	ST	CB + Control	73.22	18.19	45	GAT

control_mean	control_sd	control_n	measure	measuremax	higherisgood	cb_meanadj	control_meanadj	samplesize
0.71	0.23	81	EQ-5D	1	Yes	0.25	0.289999992	170
0.7	0.25	87	EQ-5D	1	Yes	0.25	0.300000012	185
73.2	17.9	105	SF-36 mental health subscale	100	Yes	24.39999962	26.79999924	213
73.4	20.2	95	SF-36 mental health subscale	100	Yes	24.5	26.60000038	192
63.8	19.9	133	EuroQoL	100	Yes	30	36.20000076	238
68.6	19.5	184	EuroQoL	100	Yes	29.70000076	31.39999962	342
0.664615385	0.296910691	143	EQ-5D	1	Yes	0.280000001	0.335384607	212
-6.8	13.1	56	SF-12 physical	100	No	-8.600000381	-6.800000191	184
47	11.35	187	SF-12 physical	100	Yes	53.59999847	53	562
46.4	11.25	176	SF-12 physical	100	Yes	53	53.59999847	508
56.44	15.9	45	SF-36	100	Yes	15	43.56000137	90
44.22	16.51	45	SF-36	100	Yes	26.78000069	55.77999878	90

SMD_health_ST	seSMD_health_ST	SMD_health_LT	seSMD_health_LT	_ES	_seES	_LCI	_UCI	_WT
		-0.173913002	0.15385595	-0.173913002	0.15385595	-0.475465119	0.1276391	14.36689472
-0.231756508	0.147800833							0
-0.13407819	0.137206659							0
		-0.10983216	0.144455314	-0.10983216	0.144455314	-0.392959386	0.173295066	14.58851528
		-0.326969922	0.131411985	-0.326969922	0.131411985	-0.584532678	-0.069407165	14.88309383
-0.087593123	0.108513497							0
		-0.169537604	0.146813482	-0.169537604	0.146813482	-0.457286745	0.118211515	14.53360367
		-0.144244373	0.160395771	-0.144244373	0.160395771	-0.45861432	0.170125559	14.20870781
		0.052369118	0.089536078	0.052369118	0.089536078	-0.123118371	0.227856606	15.69818497
-0.052748755	0.093255647							0
		-1.917845607	0.255622625	-1.917845607	0.255622625	-2.418856621	-1.416834474	11.72099686
-1.66951406	0.245522395							0
