# Supplementary material for: The Effectiveness of Cognitive Behavioural Treatment for Non-Specific Low Back Pain: A Systematic Review and Meta-Analysis
Source: PLoS One. 2015 Aug 5;10(8):e0134192. doi: 10.1371/journal.pone.0134192 (PMC4526658; doi:10.1371/journal.pone.0134192)
Supplement: S1 Fig — (DOCX) [file pone.0134192.s004.docx]

**Appendix S1: CENTRAL search terms (in Trials, with Back Group in Review Groups)**

#1 MeSH descriptor Back Pain explode all trees
#2 dorsalgia
#3 backache
#4 MeSH descriptor Low Back Pain explode all trees
#5 (lumbar next pain) or (coccyx) or (coccydynia) or (sciatica) or (spondylosis)
#6 MeSH descriptor Spine explode all trees
#7 MeSH descriptor Spinal Diseases explode all trees
#8 (lumbago) or (discitis) or (disc near degeneration) or (disc near prolapse) or (disc near herniation)
#9 facet near joints
#10 MeSH descriptor Intervertebral Disk explode all trees
#11 failed near back
#12 MeSH descriptor Cauda Equina explode all trees
#13 lumbar near vertebra*
#14 slipped near (disc* or disk*)
#15 degenerat* near (disc* or disk*)
#16 displace* near (disc* or disk*)
#17 prolap* near (disc* or disk*)
#18 MeSH descriptor Sciatic Neuropathy explode all trees
#19 sciatic*
#20 back disorder*
#21 back near pain
#22 COGNIT* and BEHAV*
#23 (COGNIT* or BEHAV* or CONDITIONING or RELAXATION or DESENSITI*) and (THERAP* or PSYCHOTHERAP* or TRAIN* or RETRAIN* or TREATMENT* or MODIFICATION*)
#24 DESENSITI* near PSYCHOLOG*
#25 IMPLOSIVE near THERAP*
#26 (#1 or #2 or #3 or #4 or #5 or #6 or #7 or #8 or #9 or #10 or #11 or #12 or #13 or #14 or #15 or #16 or #17 or #18 or #19 or #20 or #21) and (#22 or #23 or #24 or #25) in Other Reviews, Trials, Methods Studies, Technology Assessments, Economic Evaluations and Cochrane Groups
#27 spinal neoplasms
#28 spinal fusion
#29 postlaminectomy
#30 arachnoiditis
#31 failed near back
#32 spinal near stenosis
#33 stenosis near (spine or root or spinal)
#34 #26 not (#27 or #28 or #29 or #30 or #31 or #32 or #33) in Trials, with Back Group in Review Groups
