# Supplementary figures and images for: The Effectiveness of Cognitive Behavioural Treatment for Non-Specific Low Back Pain: A Systematic Review and Meta-Analysis
Source: PLoS One. 2015 Aug 5;10(8):e0134192. doi: 10.1371/journal.pone.0134192 (PMC4526658; doi:10.1371/journal.pone.0134192)

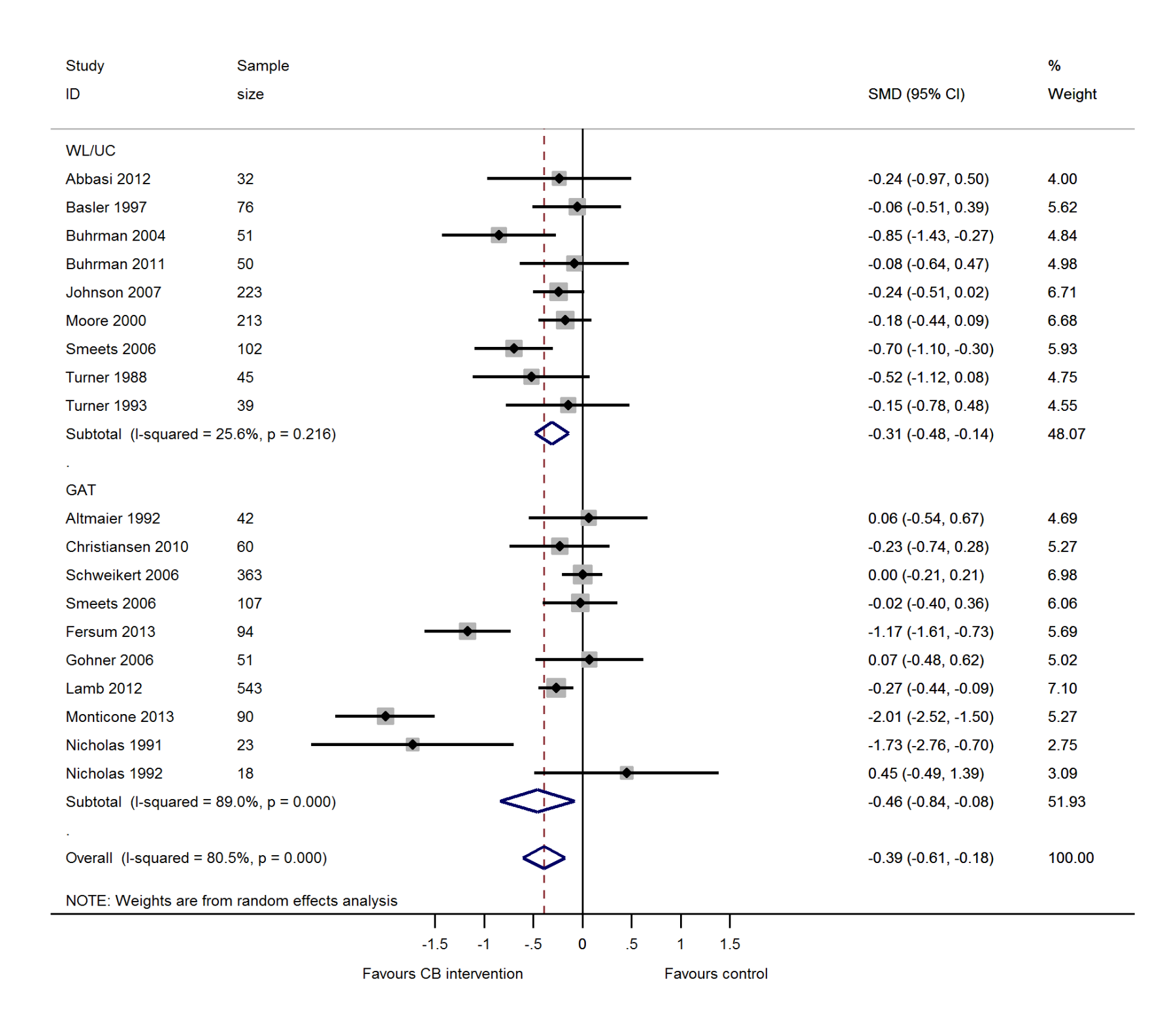

Supplement: S2 Fig — (TIF) [file pone.0134192.s005.tif]

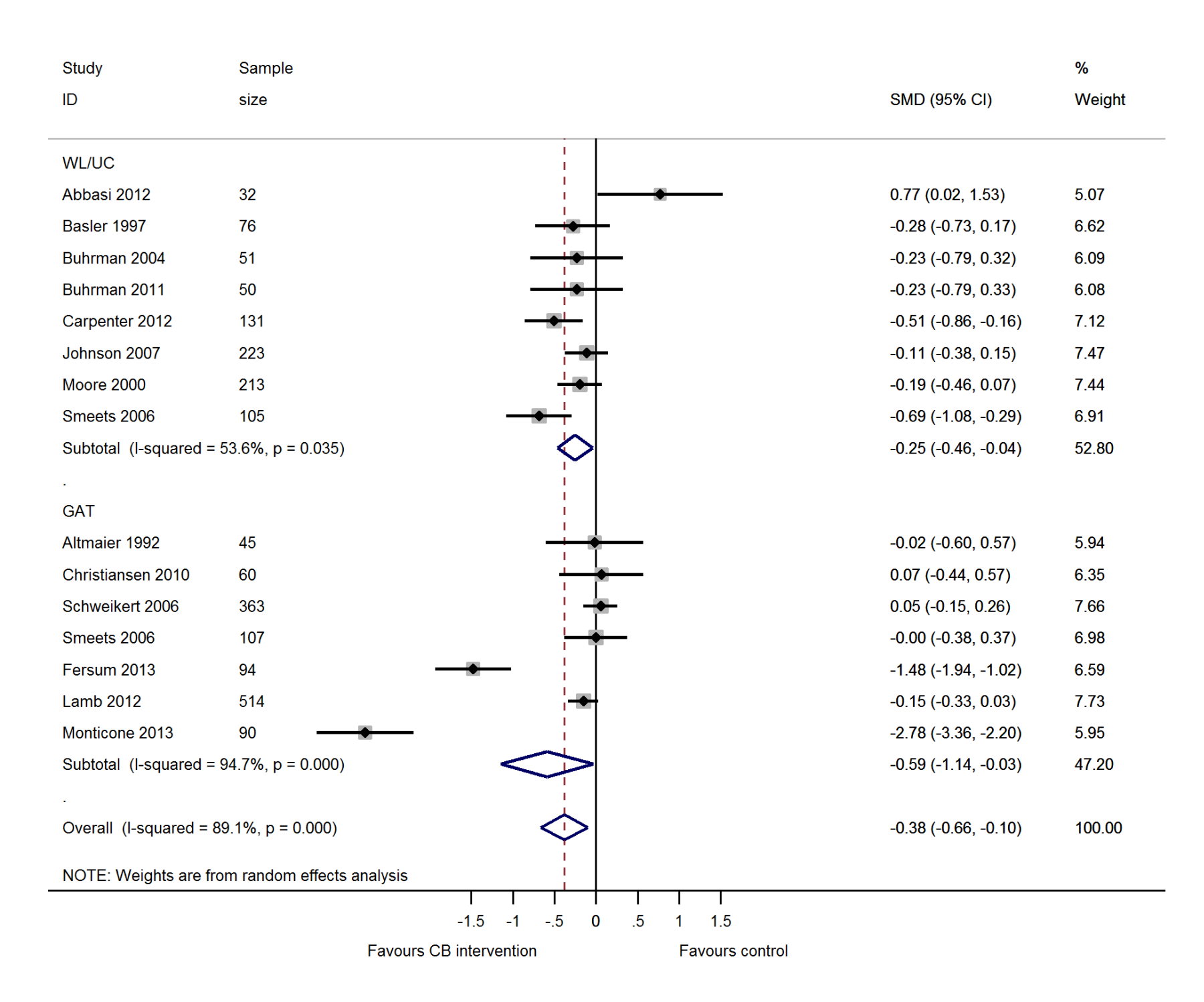

Supplement: S3 Fig — (TIF) [file pone.0134192.s006.tif]

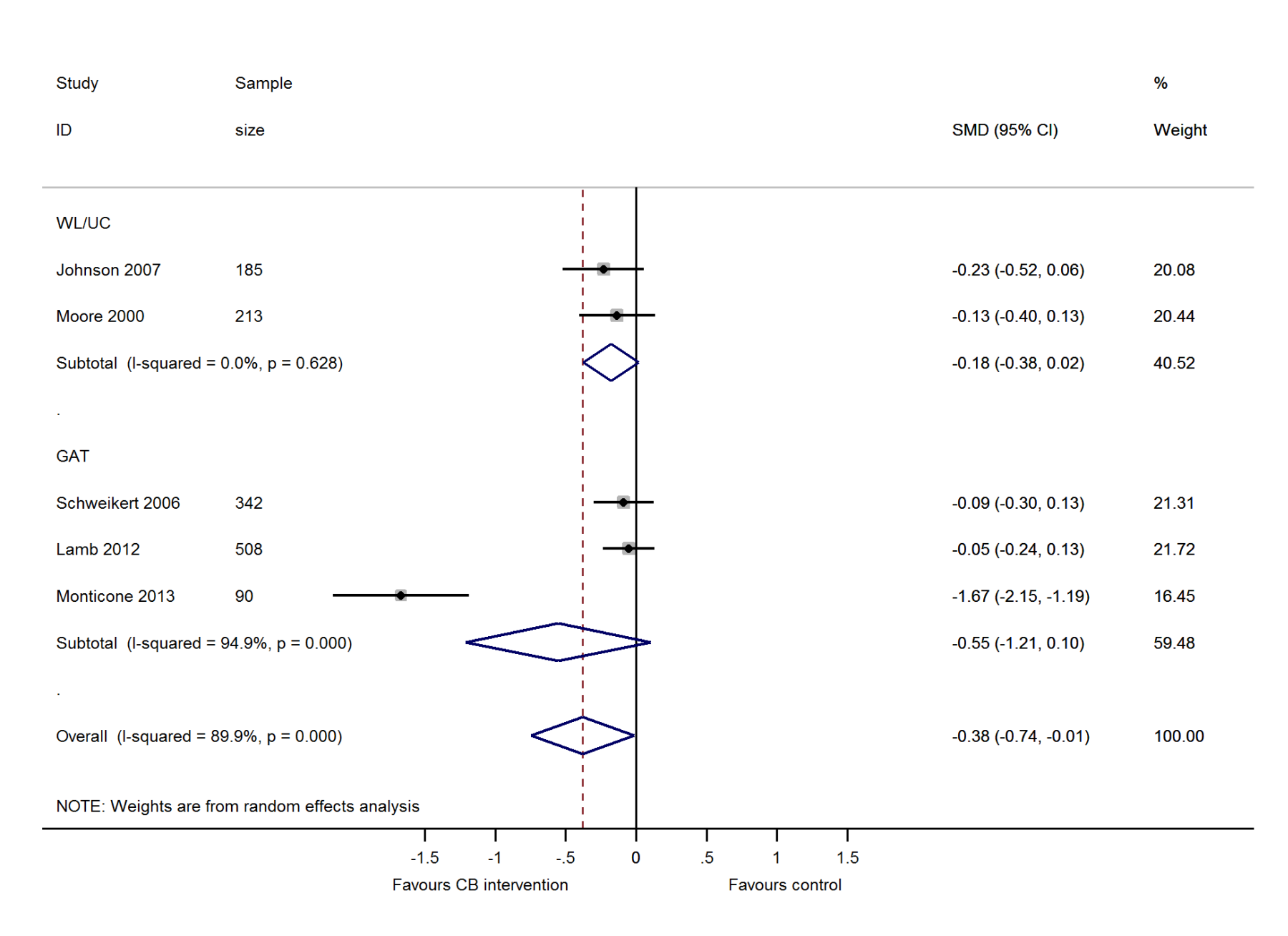

Supplement: S4 Fig — (TIF) [file pone.0134192.s007.tif]
