# Supplementary material for: The Effectiveness of Cognitive Behavioural Treatment for Non-Specific Low Back Pain: A Systematic Review and Meta-Analysis
Source: PLoS One. 2015 Aug 5;10(8):e0134192. doi: 10.1371/journal.pone.0134192 (PMC4526658; doi:10.1371/journal.pone.0134192)
Supplement: S1 Table — (DOCX) [file pone.0134192.s008.docx]

| **Study** | **Pain Duration** | **Risk of Bias** | **PRECIS Assessment** | | **GAT Type** | | | | | **GAT intensity** | |
| --- | --- | --- | --- | --- | --- | --- | --- | --- | --- | --- | --- |
|  | Months | Max 5 | GAT | CB | Home Exercise | Clinic Exercise | Out/in patient | Education/ advice | Passive modalities | Number of sessions (time per session) | Contact hours |
| Altmaier, 1992 | > 3 | 1 | E | E | No | Yes | In | Yes | ? | 3 weeks full-time | 90 |
| Christiansen, 2010 | > 6 | 2 | E | E | No | Yes | Out | Yes | Yes | 3 weeks full-time | 90 |
| Critchley, 2007 (ind)* | > 3 | 3 | P | E | Yes | No | Out | Yes | Yes | Up to 12 (30 minutes) | 6 |
| Critchley, 2007 (spn)* | > 3 | 3 | P | E | ? | Yes | Out | ? | No | Up to 8 (90 minutes) | 12 |
| Fersum, 2013 | > 3 | 1 | E | E | Yes | No | Out | No | Yes | Mean of 8 (30 minutes) | 4 |
| Gohner, 2006 | < 6 | 0 | E | E | Yes | Yes | Out | Yes | No | 6 (duration unknown) | ? |
| Hill, 2011 | ≥ 6 | 3 | P | P | Yes | ? | Out | Yes | ? | Mean of 5 (range: 3-7) (30 minutes) | 3 |
| Johnson, 2007 | ≥ 6 | 2 | P | E | No | No | Home | Yes | No | Unknown (audio tape + leaflets) | ? |
| Johnstone, 2004 | < 6 | 1 | P | E | No | Yes | Out | Yes | Yes | 6 (30 minutes) | 3 |
| Lamb, 2010 | ≥ 6 | 3 | P | E | No | No | Out | Yes | No | 1 session (15 minutes) | 0.25 |
| Monticone, 2013 | > 3 | 2 | E | P | Yes | Yes | Out | Yes | Yes | 10 (60 minutes) | 10 |
| Nicholas, 1991 | > 6 | 1 | E | P | Yes | Yes | Out | Yes | No | 10 (1 x 2 hour and 1 x 1.5 hour) | 17.5 |
| Nicholas, 1992 | > 6 | 1 | E | P | Yes | Yes | Out | Yes | No | 10 (1 x 2hour and 1 x 1.5 hour) | 17.5 |
| Schweikert, 2006 | > 6 | 1 | E | E | No | Yes | In | Yes | Yes | 3 weeks full-time | 90 |
| Smeets, 2006/8 | > 3 | 4 | E | P | No | Yes | Out | No | No | 30 (105 minutes) | 52.5 |

**Table S2. Information on GAT comparisons**

PRECIS: E = explanatory; P = pragmatic
