# Supplementary material for: The Effectiveness of Cognitive Behavioural Treatment for Non-Specific Low Back Pain: A Systematic Review and Meta-Analysis
Source: PLoS One. 2015 Aug 5;10(8):e0134192. doi: 10.1371/journal.pone.0134192 (PMC4526658; doi:10.1371/journal.pone.0134192)
Supplement: S1 Text — (PDF) [file pone.0134192.s010.pdf]

PROSPERO International prospective register of systematic reviews

Review title and timescale

1 Review title

Give the working title of the review. This must be in English. Ideally it should state succinctly the interventions or exposures being reviewed and the associated health or social problem being addressed in the review.

A systematic review of the effectiveness of cognitive behavioural treatment for non-specific low back pain

2 Original language title

For reviews in languages other than English, this field should be used to enter the title in the language of the review. This will be displayed together with the English language title.

3 Anticipated or actual start date

Give the date when the systematic review commenced, or is expected to commence.

02/06/2014

4 Anticipated completion date

Give the date by which the review is expected to be completed.

29/06/2015

5 Stage of review at time of this submission

Indicate the stage of progress of the review by ticking the relevant boxes. Reviews that have progressed beyond the point of completing data extraction at the time of initial registration are not eligible for inclusion in PROSPERO. This field should be updated when any amendments are made to a published record.

The review has not yet started ×

Review stage	Started	Completed
Preliminary searches	Yes	Yes
Piloting of the study selection process	Yes	Yes
Formal screening of search results against eligibility criteria	Yes	Yes
Data extraction	Yes	Yes
Risk of bias (quality) assessment	No	Yes
Data analysis	No	Yes

Provide any other relevant information about the stage of the review here.

Review team details

6 Named contact

The named contact acts as the guarantor for the accuracy of the information presented in the register record.

Dr Richmond

7 Named contact email

Enter the electronic mail address of the named contact.

helen.richmond@ndorms.ox.ac.uk

8 Named contact address

Enter the full postal address for the named contact.

Botnar Research Centre Nuffield Department of Orthopaedics, Rheumatology & Musculoskeletal Sciences University of Oxford Windmill Road Headington Oxford, OX3 7LD

9 Named contact phone number

Enter the telephone number for the named contact, including international dialing code.

+44 (0)1865737927

10 Organisational affiliation of the review

Full title of the organisational affiliations for this review, and website address if available. This field may be completed as 'None' if the review is not affiliated to any organisation.

University of Oxford

Website address:
http://www.ndorms.ox.ac.uk/research.php?group=rrio

11 Review team members and their organisational affiliations

Give the title, first name and last name of all members of the team working directly on the review. Give the organisational affiliations of each member of the review team.

Title	First name	Last name	Affiliation
Dr	Helen	Richmond	University of Oxford
Dr	Amanda	Hall	University of Oxford
Dr	Esther	Williamson	University of Oxford
Dr	Zara	Hansen	University of Oxford
Professor	Sallie	Lamb	University of Oxford
Miss	Bethan	Copsey	University of Oxford
Mrs	Nicolette	Hoxey-Thomas	University of Oxford
Professor	Zafra	Cooper	University of Oxford

12 Funding sources/sponsors

Give details of the individuals, organizations, groups or other legal entities who take responsibility for initiating, managing, sponsoring and/or financing the review. Any unique identification numbers assigned to the review by the individuals or bodies listed should be included.

This work is being completed as part of the NIHR Collaborations for Leadership in Applied Health Research and Care (CLAHRC).

13 Conflicts of interest

List any conditions that could lead to actual or perceived undue influence on judgements concerning the main topic investigated in the review.

Are there any actual or potential conflicts of interest?

Yes

Potential conflict of interest: Review authors have published in this field (SL, ZH) One author trains therapists in the use of a CB approach (ZH)

14 Collaborators

Give the name, affiliation and role of any individuals or organisations who are working on the review but who are not listed as review team members.

Title	First name	Last name	Organisation details

Review methods

15 Review question(s)

State the question(s) to be addressed / review objectives. Please complete a separate box for each question.

The primary objective of this systematic review is to assess the effectiveness of CB interventions, in comparison to no treatment and other conservative treatments, on patient reported pain, disability and quality of life in adults with non-specific LBP.

16 Searches

Give details of the sources to be searched, and any restrictions (e.g. language or publication period). The full search strategy is not required, but may be supplied as a link or attachment.

Cochrane Central Register of Controlled Trials (CENTRAL), MEDLINE (1966 to date), EMBASE (1988 to date), CINAHL (1982 to date), AMED (1985 to date), Physiotherapy Evidence Database (PEDro), the Cochrane Back Review Group (CBRG) Trials Register and PsycINFO. Grey literature will be searched using opensigle.

17 URL to search strategy

If you have one, give the link to your search strategy here. Alternatively you can e-mail this to PROSPERO and we will store and link to it.

I give permission for this file to be made publicly available
Yes

18 Condition or domain being studied
Give a short description of the disease, condition or healthcare domain being studied. This could include health and wellbeing outcomes.
The condition being studied is low back pain of any duration in adults. Relevant outcomes are: pain, disability, quality of life, function, work-disability and/or cost.

19 Participants/population
Give summary criteria for the participants or populations being studied by the review. The preferred format includes details of both inclusion and exclusion criteria.
Inclusion: Adult participants (males and females over the age of 18) with a clinical diagnosis of non-specific LBP +/- radiating leg pain. Exclusion: Participants with a pathological cause of LBP, such as, infection, neoplasm, metastasis, osteoporosis, rheumatoid arthritis, fractures, spinal canal stenosis, or nerve root compromise. Participants with neurodegenerative conditions (such as, multiple sclerosis), or women experiencing LBP during pregnancy, will also be excluded.

20 Intervention(s), exposure(s)
Give full and clear descriptions of the nature of the interventions or the exposures to be reviewed
Intervention: Cognitive behaviour therapy (CBT) delivered by any health professional. CBT interventions will be included if the intervention meets the following two criteria: a) is explicitly or implicitly based on the CB model (where the use of CBT/CB in relation to the intervention is explicitly stated OR where the connection between thoughts, feelings and behaviours in relation to the intervention is implicitly described); and b) uses specific techniques to both change cognitions and change behaviours. Psychological interventions that are not explicitly or implicitly based on the CB model will be excluded. Interventions using techniques to change either cognitions or behaviours, but not both, will also be excluded. Interventions delivered by lay personnel will be excluded. In cases where treatments are multimodal, for example, including CB as a component of a comprehensive back school, the intervention will be deemed eligible only when the main focus of the intervention was based on CB.

21 Comparator(s)/control
Where relevant, give details of the alternatives against which the main subject/topic of the review will be compared (e.g. another intervention or a non-exposed control group).
We will explore the effects of CB in relation to the following comparison conditions: No treatment (WL/UC): A trial arm in which participants received no active treatment during the study period, this included studies with a wait-list (WL) comparison or a comparison defined as usual care (UC) in which no prescribed treatment was provided within the trial. Guideline-based Active Treatment (GAT): A prescribed/supervised treatment in line with the European Guidelines (2009). A trial arm in which participants were allocated to receive an active treatment, in line with the European LBP guidelines, the details of which were specified in some way. Studies comparing different types of CB intervention (e.g. one to one versus group interventions) will only be included where a non-CB control arm is included. Studies comparing CB interventions to a surgical comparator will be excluded. Studies comparing a CB intervention to a drug based comparison will only be included where the drug type and dosage are in line with the current European LBP guidelines (2009).

22 Types of study to be included initially
Give details of the study designs to be included in the review. If there are no restrictions on the types of study design eligible for inclusion, this should be stated.
Only randomised controlled trials (RCTs) will be included. Non-randomised and quasi-randomised trials will be excluded.

23 Context
Give summary details of the setting and other relevant characteristics which help define the inclusion or exclusion criteria.

24 Primary outcome(s)
Give the most important outcomes.
Pain and/or condition specific disability

Give information on timing and effect measures, as appropriate.
Data will be classified according to the following time points: • Short Term (ST): as close to 6 weeks, not exceeding

12 weeks • Long Term (LT): as close to 52 weeks, no less than 26 weeks (if 2 time points of 6 months or more, closest to 52 weeks will be used)

25 Secondary outcomes

List any additional outcomes that will be addressed. If there are no secondary outcomes enter None.
Generic health status/Quality of life Work disability Economic/cost-effectiveness

Give information on timing and effect measures, as appropriate.

26 Data extraction, (selection and coding)

Give the procedure for selecting studies for the review and extracting data, including the number of researchers involved and how discrepancies will be resolved. List the data to be extracted.
All study titles and abstracts retrieved from the literature searches will be independently assessed for eligibility by two review authors. Studies deemed potentially eligible, or where there is insufficient information to determine eligibility from the title and abstract, will be obtained in full and compared against the inclusion criteria for this review. Three review authors (AH, HR and BC) will independently extract study data using a standardised form, adapted from the example form provided on the Cochrane Back Review Group (CBRG (2013a) website. The following information will be extracted from each RCT: • Trial patient characteristics (including total number randomised to each intervention group; mean age, country, and duration of symptoms. • Intervention and comparison (including intervention and comparison treatment details including duration, dose, mode, provider (profession), and details of any co-interventions). • Outcomes (including baseline demographics, any of the pre-specified primary or secondary outcomes, and any blinding) • Trial results (including attrition; method of analysis, numbers analysed in all groups; mean change and standard deviation of relevant outcome measures in the intervention and comparison groups and attrition). When available, multiple published sources will be checked for each study, in order to provide the reviewers with the most amount of information.

27 Risk of bias (quality) assessment

State whether and how risk of bias will be assessed, how the quality of individual studies will be assessed, and whether and how this will influence the planned synthesis.
The risk of bias for each article will be independently assessed by two reviewers (NT and HR). Risk of bias will be assessed against the updated Cochrane CBRG criteria which classifies risk of bias into 6 domains (selection bias, performance, bias, detection bias, attritions bias, reporting bias and other bias) (Higgins 2011 BMJ). For each study, the domains will be rated as “low”, “high” or “unclear” according to Cochrane’s criteria for judging risk of bias (handbook.cochrane.org, section 8.5d). A consensus method will be used to come to a conclusion about the risk of bias of included studies. However, if agreement is not achieved at any stage, a further review author (EW, BC or AH) will be consulted. If either of the review authors are a (co-) author of one of the included studies, they will not be involved in the assessment for the risk of bias of that trial in this review. If risk of bias for an included study is previously assessed in a Cochrane review, these assessments will be used.

28 Strategy for data synthesis

Give the planned general approach to be used, for example whether the data to be used will be aggregate or at the level of individual participants, and whether a quantitative or narrative (descriptive) synthesis is planned. Where appropriate a brief outline of analytic approach should be given.
Follow-up time points: • Short Term (ST): as close to 6 weeks, not exceeding 12 weeks • Long Term (LT): as close to 52 weeks, no less than 26 weeks (if 2 time points of 6 months or more, closest to 52 weeks will be used) Primary Contrast: We are interested in the effect of CB versus any of the aforementioned comparisons. However, we recognise that the comparison arms may be clinically heterogeneous in terms of treatment prescription and hence a high level of statistical heterogeneity is anticipated, making the result of an overall comparison difficult to interpret, thus we will stratify the analysis based on the type of comparison group. While we will analyse the data at a short-term time-point (closely aligned with the end of the intervention) to report the immediate effects of CB compared to other treatments, our primary endpoint for establishing effect is at the long-term time point. We have chosen a long-term time-point as our primary endpoint because it is of particular relevance for healthcare policy and clinicians to understand if the effects of CB are maintained over a longer time period. Any improvement for less than 6 months could be argued to be of little clinical value. The primary contrast is thus: • CB +/- any included comparison arm vs the comparison arm at long-term follow-up (between 6-12 months) Stratified by: • Comparison Arm (WL/UC; GAT) Where a study contains either two eligible control or intervention groups and one intervention or control group, the two eligible groups will be combined to get one effect size for the study. This is to avoid double-counting the intervention group and biasing any meta-analyses.

Review general information

30 Type of review

Select the type of review from the drop down list.
Intervention

31 Language
Select the language(s) in which the review is being written and will be made available, from the drop down list. Use the control key to select more than one language.
English

Will a summary/abstract be made available in English?
Yes

32 Country
Select the country in which the review is being carried out from the drop down list. For multi-national collaborations select all the countries involved. Use the control key to select more than one country.
England

33 Other registration details
Give the name of any organisation where the systematic review title or protocol is registered together with any unique identification number assigned. If extracted data will be stored and made available through a repository such as the Systematic Review Data Repository (SRDR), details and a link should be included here.

34 Reference and/or URL for published protocol
Give the citation for the published protocol, if there is one.
Give the link to the published protocol, if there is one. This may be to an external site or to a protocol deposited with CRD in pdf format.

I give permission for this file to be made publicly available
Yes

35 Dissemination plans
Give brief details of plans for communicating essential messages from the review to the appropriate audiences.
Dissemination at relevant conferences, publication in peer reviewed journal, and active dissemination with local departments and national stakeholders.

Do you intend to publish the review on completion?
Yes

36 Keywords
Give words or phrases that best describe the review. (One word per box, create a new box for each term)
Back

Pain

Cognitive

Behaviour

Therapy

37 Details of any existing review of the same topic by the same authors
Give details of earlier versions of the systematic review if an update of an existing review is being registered, including full bibliographic reference if possible.

38 Current review status
Review status should be updated when the review is completed and when it is published.
Ongoing

39 Any additional information
Provide any further information the review team consider relevant to the registration of the review.

40 Details of final report/publication(s)
This field should be left empty until details of the completed review are available.
Give the full citation for the final report or publication of the systematic review.

Give the URL where available.
